# Critical Role of Iodous Acid in Neutral Iodine Oxoacid Nucleation

**DOI:** 10.1021/acs.est.2c04328

**Published:** 2022-09-20

**Authors:** Rongjie Zhang, Hong-Bin Xie, Fangfang Ma, Jingwen Chen, Siddharth Iyer, Mario Simon, Martin Heinritzi, Jiali Shen, Yee Jun Tham, Theo Kurtén, Douglas R. Worsnop, Jasper Kirkby, Joachim Curtius, Mikko Sipilä, Markku Kulmala, Xu-Cheng He

**Affiliations:** † Key Laboratory of Industrial Ecology and Environmental Engineering (Ministry of Education), School of Environmental Science and Technology, Dalian University of Technology, Dalian 116024, China; ‡ Aerosol Physics Laboratory, Faculty of Engineering and Natural Sciences, Tampere University, Tampere 33014, Finland; § Institute for Atmospheric and Environmental Sciences, Goethe University Frankfurt, Frankfurt am Main 60438, Germany; ∥ Institute for Atmospheric and Earth System Research/Physics, Faculty of Science, University of Helsinki, Helsinki 00014, Finland; ⊥ School of Marine Sciences, Sun Yat-sen University, Zhuhai 519082, China; # Department of Chemistry, University of Helsinki, Helsinki 00014, Finland; ∇ Aerodyne Research, Inc., Billerica, Massachusetts 01821, United States; ○ CERN, the European Organization for Nuclear Research, CH-1211 Geneva 23, Switzerland; ◆ Joint International Research Laboratory of Atmospheric and Earth System Sciences, School of Atmospheric Sciences, Nanjing University, Nanjing 210023, China; ¶ Aerosol and Haze Laboratory, Beijing Advanced Innovation Center for Soft Matter Science and Engineering, Beijing University of Chemical Technology, Beijing 100029, China; & Center for Atmospheric Particle Studies, Carnegie Mellon University, Pittsburgh, Pennsylvania 15213, United States

**Keywords:** quantum chemical calculation, particle formation, atmospheric cluster dynamics simulation, iodic acid, iodous acid, iodine oxoacid nucleation

## Abstract

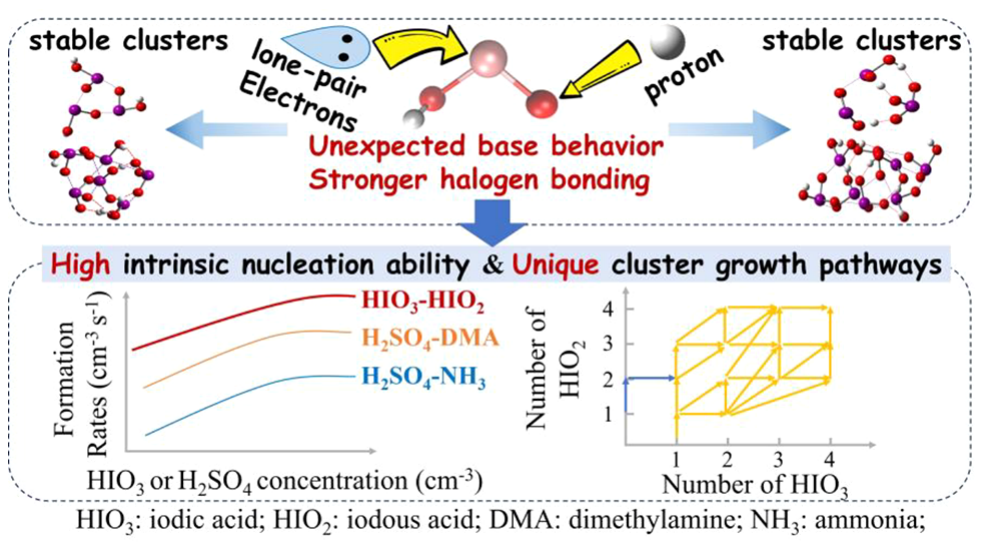

Nucleation of neutral iodine particles has recently been found to involve both iodic acid (HIO_3_) and iodous acid (HIO_2_). However, the precise role of HIO_2_ in iodine oxoacid nucleation remains unclear. Herein, we probe such a role by investigating the cluster formation mechanisms and kinetics of (HIO_3_)_*m*_(HIO_2_)_*n*_ (*m* = 0–4, *n* = 0–4) clusters with quantum chemical calculations and atmospheric cluster dynamics modeling. When compared with HIO_3_, we find that HIO_2_ binds more strongly with HIO_3_ and also more strongly with HIO_2_. After accounting for ambient vapor concentrations, the fastest nucleation rate is predicted for mixed HIO_3_–HIO_2_ clusters rather than for pure HIO_3_ or HIO_2_ ones. Our calculations reveal that the strong binding results from HIO_2_ exhibiting a base behavior (accepting a proton from HIO_3_) and forming stronger halogen bonds. Moreover, the binding energies of (HIO_3_)_*m*_(HIO_2_)_*n*_ clusters show a far more tolerant choice of growth paths when compared with the strict stoichiometry required for sulfuric acid–base nucleation. Our predicted cluster formation rates and dimer concentrations are acceptably consistent with those measured by the Cosmic Leaving Outdoor Droplets (CLOUD) experiment. This study suggests that HIO_2_ could facilitate the nucleation of other acids beyond HIO_3_ in regions where base vapors such as ammonia or amines are scarce.

## Abstract 2

The unexpected base behavior and stronger halogen bonding of iodous acid (HIO_2_) are shown to be responsible for the critical role of HIO_2_ in iodine oxoacid nucleation, warranting further studies on a potentially wider role of HIO_2_ in nucleation of marine atmospheres.

## Introduction

New particle formation (NPF) contributes to more than half of the global cloud condensation nuclei, which in turn contribute to cloud formation.^1−4^ Therefore, NPF ultimately affects climate change.^5,6^ Compared to clouds over land, marine clouds play a larger role in the climate system not only due to their wider coverage but also because they significantly increase the albedo of oceans.^7−9^ Hence, understanding marine particle formation processes is essential. Sulfuric acid (SA, H_2_SO_4_) and methane sulfonic acid (MSA, CH_3_HSO_3_) are commonly thought to contribute to marine particle formation.^10−15^ In the critical initial clusters during nucleation, SA and MSA molecules are stabilized by base molecules such as ammonia (NH_3_) and amines (e.g., dimethylamine (DMA)).^16−25^

Besides SA and MSA, iodine-containing molecules were proposed to account for particle bursts observed over 20 years ago in coastal regions at the Mace Head Observatory, Ireland.^26,27^ Iodine dioxide (OIO) was among the first candidates proposed to account for this rapid particle formation, and OIO was believed to form stable iodine tetroxide (I_2_O_4_) in the particles.^27,28^ However, a following study examined the composition and morphology of iodine-containing particles and observed iodine pentoxide (I_2_O_5_) as the primary constituent of these particles.^29^ Subsequent laboratory investigations alternatively proposed iodine oxides, e.g., I_2_O_*y*_ (*y* = 3–5) as the critical vapors initializing iodine particle formation, while the restructuring of these iodine oxides in the particle phase contributed to the observed O/I ratio of 2.5.^29−31^ However, recent measurements with a nitrate chemical ionization mass spectrometer (nitrate-CIMS) revealed extremely high concentrations of iodic acid (HIO_3_), occasionally above 10^8^ cm^–3^, at the Mace Head Observatory.^32^ Such high concentrations of HIO_3_ lead to rapid particle formation.^32−35^ In contrast to ambient observations, recent laboratory studies with high iodine concentrations shed doubts on this mechanism and proposed that I_2_O_*y*_ could have been interpreted as gaseous HIO_3_.^36,37^

Sophisticated experiments were carried out in the Cosmic Leaving Outdoor Droplets (CLOUD) chamber at CERN to study iodine particle formation at atmospherically relevant conditions to resolve the puzzles. With a finely tuned nitrate-CIMS, gaseous HIO_3_ was unambiguously measured.^35^ By initializing an ion-induced nucleation experiment from ion-free conditions and tracing the subsequent development of charged iodine clusters, the authors obtained the time evolution of charged iodine clusters containing up to 11 iodine atoms.^35,38^ The sequential charged clusters differ by the addition of a single HIO_3_ molecule and cannot be explained by any molecule containing two iodine atoms (I_2_O_*y*_), confirming an earlier finding by Sipilä et al.^32^ On the other hand, neutral iodine nucleation was found to proceed through a novel iodic acid (HIO_3_)–iodous acid (HIO_2_) mechanism.^35^ The particle growth was primarily contributed by HIO_3_, while the I_2_O_4_ concentration, at ca. 1% HIO_3_, was too low to make a significant contribution.^35^ In contrast to SA and bases such as NH_3_ and DMA, which can be independently controlled in the laboratory, iodine oxoacids (HIO_2–3_) originate from the same precursor, e.g., elemental iodine,^36,39^ and so it is difficult to separate their roles in atmospheric particle formation. This poses challenges to determining the relative importance of the three channels: (1) pure HIO_3_, (2) mixed HIO_3_ and HIO_2_, and (3) pure HIO_2_ nucleation of neutral iodine oxoacids (defined as the sum of the three channels).

Besides laboratory experiments and field observations, quantum chemical calculations have also been used to predict iodine particle formation mechanisms. In polluted locations, SA, MSA, and NH_3_ were suggested to enhance pure HIO_3_ nucleation.^14,40,41^ However, so far, the predicted nucleation rates for (1) pure HIO_3_, (2) HIO_3_–SA, (3) HIO_3_–MSA, and (4) HIO_3_–NH_3_ cannot account for the experimental results on pure iodine nucleation from CLOUD.^35^ One likely reason for such discrepancies is that earlier studies considered only the sequential addition of HIO_3_ and did not include HIO_2_.^32^ Very recently, Zhang et al. investigated the nucleation of pure HIO_2_ and found that the cluster formation rate of HIO_2_ is faster than that of pure HIO_3_,^42^ yet remaining lower than the CLOUD measurements.^35^

To evaluate the role of HIO_2_ in neutral iodine oxoacid nucleation, we use quantum chemical calculations to optimize the geometries of (HIO_3_)_*m*_(HIO_2_)_*n*_ (*m* = 0–4, *n* = 0–4) clusters and calculate corresponding thermodynamic data, which in turn are used as inputs for the Atmospheric Cluster Dynamics Code (ACDC) model to probe the cluster formation mechanisms and kinetics.^43^ Furthermore, we provide a comparison of neutral iodine oxoacid nucleation with the benchmarks of neutral SA–DMA/NH_3_ nucleation under similar conditions to gauge the potential atmospheric importance of iodine oxoacid nucleation.

## Computational Framework

### Quantum Chemical Calculations

Here, a multistep global minimum sampling scheme was employed to search for the global minima of (HIO_3_)_*m*_(HIO_2_)_*n*_ (*m* = 0–4, *n* = 1–4) clusters with additional geometries of (HIO_3_)_1–4_ clusters adopted from previous studies.^40,44^ We used an in-house code to generate 3000–5000 initial configurations for each cluster with *n* molecules by randomly placing a new molecule around cluster minima with *n* – 1 molecules. The initial configurations were further optimized at the semiempirical PM7 level of theory.^45^ Single-point energy calculations at the M06-2X/def2-TZVP level of theory were subsequently performed on all the optimized geometries. Additional optimizations and frequency calculations of conformers within 10–15 kcal mol^–1^ higher energy compared to the identified lowest energy conformer were performed at the M06-2X/Basis1 (Basis1 presents 6-31++G(d,p) for H and O atoms and aug-cc-pVTZ-PP with ECP28 for I atoms^46^) level of theory. If the geometry optimization failed or there were imaginary frequencies for the optimized conformers, the input geometries will be adjusted and re-optimized until a “successful” optimization without imaginary frequencies was obtained. Single-point energy calculations at the DLPNO-CCSD(T)/Basis2 (Basis2 presents aug-cc-pVTZ for H and O atoms and aug-cc-pVTZ-PP with ECP28 for I atoms) level of theory were further performed on selected low-free energy conformers optimized at the M06-2X/Basis1 level of theory. Similar to previous studies,^47^ we employed the GoodVibes program^48^ to recalculate the Gibbs free energy correction term (via quasi-harmonic correction) of (HIO_3_)_*m*_(HIO_2_)_*n*_ (*m* = 0–4, *n* = 0–4) clusters at the M06-2X/Basis1 level to decrease the possible error caused by the rigid-rotor-harmonic-oscillator approximation. We used 100 cm^–1^ as the low-frequency cutoff value. Finally, the conformer with the lowest Gibbs free energy at 298.15 K (combining the single-point energies at the DLPNO-CCSD(T)/Basis2 level and the recalculated Gibbs free energy correction terms by GoodVibes) was selected as the global minimum for a given cluster. We note that mixing basis sets of different sizes (e.g., in Basis1, 6-31++G(d,p) was used for H and O atoms and aug-cc-pVTZ-PP with ECP28 for I atoms) could, in some cases, lead to substantial errors but, in our case, test calculations demonstrate that such mixture has a minimal effect on the calculated formation free energy (Δ*G*) (see the test results in Table S1). In addition, Gibbs free energies at other temperatures were obtained by combining the single-point energies at the DLPNO-CCSD(T)/Basis2 level and the recalculated Gibbs free energy correction terms by GoodVibes at the corresponding temperature. All geometry optimization, vibrational frequency calculations, and single-point energies using the PM7 and M06-2X methods were performed in the GAUSSIAN 16 program package,^49^ and DLPNO-CCSD(T)/Basis2 calculations were performed using the ORCA 4.0.0 program^50^ with tight SCF and PNO convergence criteria. The pure (HIO_3_)_1–4_ clusters from previous studies^40,44^ were re-optimized, followed by single-point energy calculations at the same theory levels of this study. The Δ*G* values for individual clusters were obtained by subtracting the sum of Gibbs free energies of their constituent molecules from that of the clusters at the considered temperature.

### ACDC Modeling

We employed the ACDC model to study the time evolutions of cluster formation rates, steady-state concentrations, and growth pathways of clusters.^43^ The detailed description of the ACDC can be found in a previous study,^43^ and we present the physical principles of ACDC in the Supporting Information (SI). In this study, the simulated clusters are (HIO_3_)_*m*_(HIO_2_)_*n*_ (*m* = 0–4, *n =* 0–4); i.e., the maximum number of HIO_3_ and HIO_2_ molecules in the system is four of each. The diameter of the largest cluster ((HIO_3_)_4_(HIO_2_)_4_) is around 1.5 nm, which is calculated in Multiwfn version 3.7^51^ by the maximum distance between two atoms considering their van der Waals radii. The size of the largest cluster is comparable to the 1.7 nm for the nucleation rates reported in the CLOUD experiment.^35^ The (HIO_3_)_5_(HIO_2_)_4_ and (HIO_3_)_4_(HIO_2_)_5_ clusters are set as the boundary clusters, which are allowed to grow out of the system and contribute to the cluster formation rate (see details in the SI). In the simulation, the HIO_3_ concentration [HIO_3_] was set to between 10^6^ and 10^8^ cm^–3^ and the HIO_2_ concentration [HIO_2_] between 10^4^ and 10^6^ cm^–3^, corresponding to ambient concentrations.^32,35^ The simulations were mainly carried out at 263.15 K (−10 °C), employing a constant coagulation sink (CS) coefficient of 2 × 10^–3^ s^–1^, a typical value at coastal regions^52^ and similar to the CLOUD wall loss rate. In addition, a smaller CS value of 2 × 10^–4^ s^–1^, corresponding to the case of clean atmosphere over the Arctic Ocean,^33^ and a larger CS value of 2 × 10^–2^ s^–1^, corresponding to the case of urban and polluted atmosphere,^53^ were selected to test the effects of the CS on the nucleation rates. To make a direct comparison with CLOUD measurements, the simulations were also run under the same precursor concentrations (Table S2) and wall loss rates (Table S3) for each cluster and temperature (+10 and −10 °C). In ACDC, the collision rate coefficients were calculated by the hard sphere kinetic gas theory.^43^ Previous studies have found that the actual collision coefficient is additionally enhanced by attractive van der Waals forces (e.g., dipole–dipole interaction and dispersion interaction).^54,55^ Here, the enhancement factor for the iodine oxoacid system was approximately estimated to be 2.4 based on the dipole–dipole interaction or dispersion interaction (see details in the SI). For ACDC modeling of pure HIO_3_ and pure HIO_2_ nucleation, the (HIO_3_)_5_ and (HIO_2_)_5_ clusters, respectively, were set as boundary clusters, with the remaining parameterizations identical to those of the HIO_3_–HIO_2_ nucleation.

## Results and Discussion

### Cluster Structures

**Figure 1:**
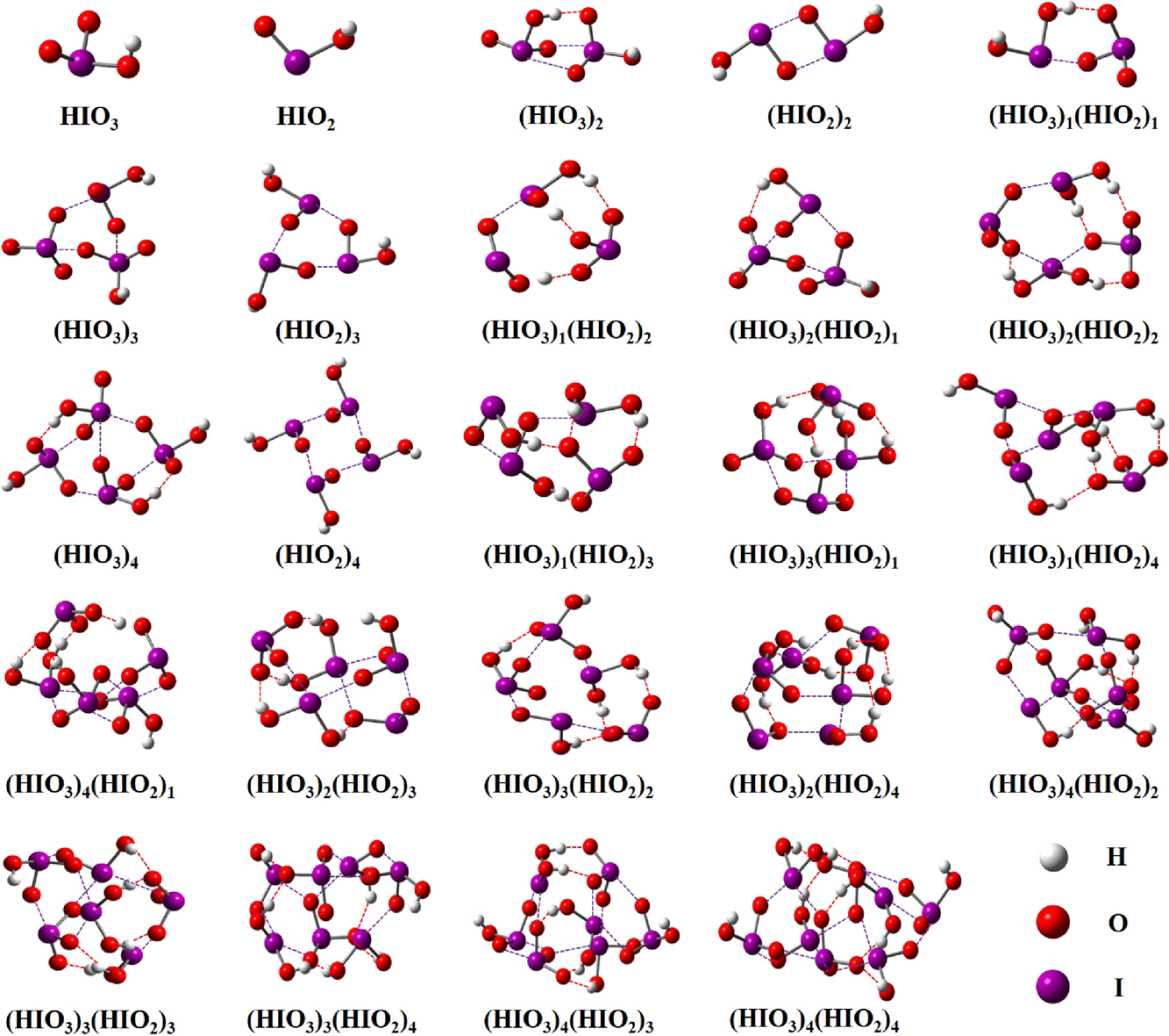
Lowest formation free energy conformers of the (HIO_3_)_*m*_(HIO_2_)_*n*_ (*m* = 0–4, *n* = 0–4) clusters calculated at the DLPNO-CCSD(T)/Basis2//M06-2X/Basis1 level of theory. The dashed red lines indicate HBs. The dashed purple lines indicate XBs.

The global minimum structures of (HIO_3_)_*m*_(HIO_2_)_*n*_ (*m* = 0–4, *n* = 0–4) clusters are presented in Figure 1. The geometries of homomolecular (HIO_3_)_1–4_ clusters are adopted from previous studies,^40,44^ while the rest are searched and calculated in this study. It deserves mentioning that there are four reported (HIO_3_)_2_ conformers;^35,40,44,56^ we used the one from Kumar et al.,^44^ which has the lowest Δ*G* (see details for geometries and Δ*G* values in Table S4). A common feature for all the clusters is that halogen bonds (O–I···O bond, herein denoted as XB) or together with hydrogen bonds (O–H···O bond, herein denoted as HB) are formed. Interestingly, proton transfer reactions are observed in all (HIO_3_)_*m*_(HIO_2_)_*n*_ (*m* = 1–4, *n* = 1–4) clusters except (HIO_3_)_2_(HIO_2_)_1_, while no proton transfer is observed in any pure HIO_2_ and HIO_3_ clusters. It deserves mentioning that the observed proton transfer is a spontaneous process. The spontaneous proton transfer was confirmed by re-optimizing the “proton-returned” conformer. The “proton-returned” conformer was manually built by pulling the proton back to the original location and increasing the distance between the two molecules. After the re-optimization, proton transfer can still occur, indicating a spontaneous process.

For most of (HIO_3_)_*m*_(HIO_2_)_*n*_ (*m* = 1–4, *n* = 1–4) clusters, the proton is transferred from HIO_3_ to HIO_2_. Therefore, HIO_2_ behaves as a Brønsted–Lowry base when interacting with HIO_3_. To the best of our knowledge, this is the first time that HIO_2_ is revealed to behave as a base in the interaction with HIO_3_. Previous studies have found that the acidity of HIO_3_ (acid dissociation constant, p*K*_a_ = 0.80)^57^ is much higher than that of HIO_2_ (p*K*_a_ = 6),^58^ supporting our observations. Surprisingly, proton transfer can also occur between two HIO_2_ molecules in (HIO_3_)_2_(HIO_2_)_3_ and (HIO_3_)_2_(HIO_2_)_4_ clusters. In these cases, the HIO_2_ molecules acting as proton acceptor and donor have distinct interactions with their adjacent molecules: (1) the proton acceptor HIO_2_ forms two XBs via its I atom with adjacent molecules, and (2) the proton donor HIO_2_ forms two XBs via its two O atoms with adjacent molecules, while its I atom does not form additional bonds with other molecules. These surprising characteristics therefore result from certain interactions of an HIO_2_ molecule with its adjacent molecules that serve to modify the effective HIO_2_ acidity.

### Cluster Formation Free Energy

**Figure 2:**
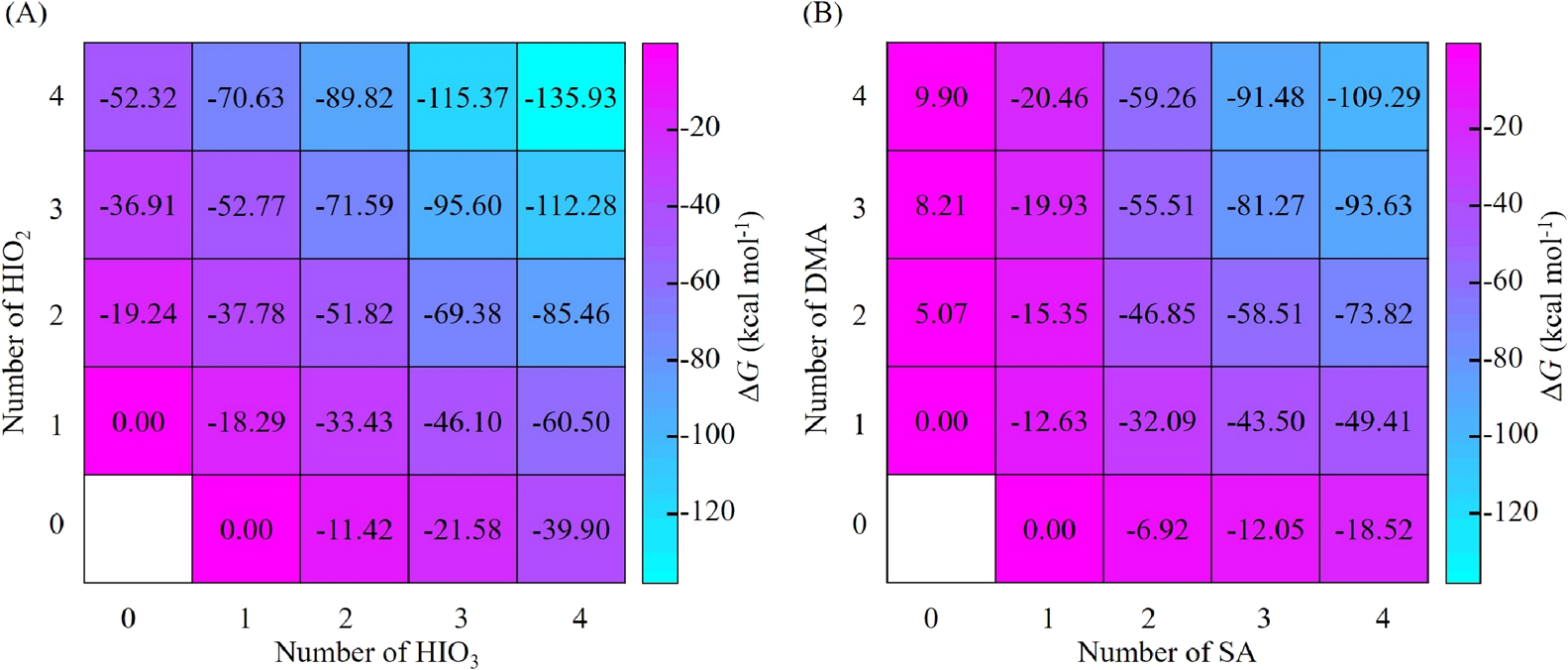
Formation free energy (Δ*G*) with quasi-harmonic correction of (A) (HIO_3_)_*m*_(HIO_2_)_*n*_ and (B) (SA)_*m*_(DMA)_*n*_ (adopted from Xie et al.^60^) clusters (*m* = 0–4, *n* = 0–4) calculated at the DLPNO-CCSD(T)/Basis2//M06-2X/Basis1 and DLPNO-CCSD(T)/aug-cc-pVTZ//ωB97X-D/6-31++G(d,p) levels, respectively. The calculations are performed at 263.15 K and 1 atm.

We present the formation free energy surface with quasi-harmonic correction at 263.15 K for the iodine oxoacid system in Figure 2A, with the corresponding one at 298.15 K presented in Figure S1. Previous studies have shown that SA–DMA-driven NPF is dominant in the urban atmosphere^53^ and that it almost proceeds at the SA kinetic limit.^59^ Here, the Δ*G* values of the SA–DMA system (Figure 2B)^60^ are used as a benchmark to compare with the calculated Δ*G* values of the iodine oxoacid system to show the effectiveness of HIO_3_–HIO_2_ cluster formation. The Δ*G* value of each of the HIO_3_–HIO_2_ clusters is lower than that of the corresponding SA–DMA clusters, with the difference in their Δ*G* values varying between 1.34 and 62.21 kcal mol^–1^. Such a large difference in Δ*G* values for all clusters indicates that iodine oxoacid cluster formation is thermodynamically even more favorable than SA–DMA cluster formation, which has hitherto represented one of the most efficiently known neutral nucleating mechanisms observed in the atmosphere. In addition, the Δ*G* values for the (HIO_3_)_*m*_(HIO_2_)_*n*_ (*m* < *n*) clusters above the diagonal line are lower than those of the corresponding (HIO_3_)_*m*_(HIO_2_)_*n*_ (*m* > *n*) clusters below the diagonal line, representing a reverse trend compared with the case of the SA–DMA system.^60^ The lower Δ*G* values for HIO_2_-rich clusters indicate a stronger binding ability of HIO_2_ compared with that of HIO_3_, confirming the important role of HIO_2_ in iodine oxoacid nucleation.

Since dimer formation is the critical first step of cluster formation and the dimer contains the simplest interaction between two monomers, the Δ*G* values and interaction patterns of (HIO_2_)_2_, (HIO_3_)_2_, and (HIO_3_)_1_(HIO_2_)_1_ are further analyzed here. As can be seen in Figure 2A, Δ*G* values decrease in the order (HIO_3_)_2_ (−11.42 kcal mol^–1^) > (HIO_3_)_1_(HIO_2_)_1_ (−18.29 kcal mol^–1^) > (HIO_2_)_2_ (−19.24 kcal mol^–1^). Therefore, HIO_2_ has a stronger ability to bind with HIO_3_ and HIO_2_, compared with HIO_3_, in agreement with the observation that HIO_2_-rich clusters are more stable. It deserves mentioning that the Δ*G* of the identified (HIO_3_)_1_(HIO_2_)_1_ is lower than previously reported one^44^ and the identified (HIO_2_)_2_ is the same as that in a previous study^42^ (see details for geometries and Δ*G* values in Table S4). As can be seen in Figure 1, (HIO_2_)_2_ contains only two XBs, while (HIO_3_)_1_(HIO_2_)_1_ contains proton-transfer-induced electrostatic attraction plus one HB and one XB, and (HIO_3_)_2_ contains two XBs and one HB. Therefore, the XB strength between two HIO_2_ is much stronger than that between two HIO_3_, indicating the higher XB formation ability of HIO_2_ compared with HIO_3_ in the formation of (HIO_3_)_*m*_(HIO_2_)_*n*_ (*m* = 1–4, *n* = 1–4). The stronger XB strength between two HIO_2_ compared with that between two HIO_3_ is supported by their shorter XB bond length and smaller energy gap between antibonding orbital δ* (O–I) and lone-pair orbital LP(O), which are two critical molecular orbitals for forming XB (Table S5). We also located the (HIO_3_)_1_(HIO_2_)_1_ conformer with only two XBs, which has higher Δ*G* than the global minimum with proton-transfer-induced electrostatic interaction, one HB and one XB. This indicates that proton-transfer-induced electrostatic attraction plays a more important role than XB in the formation of (HIO_3_)_1_(HIO_2_)_1_, highlighting the critical role of the basicity of HIO_2_. All in all, the basicity of HIO_2_ and the stronger XB formation ability together explain the key role of HIO_2_ in the iodine oxoacid nucleation.

### Evaporation Rates

**Figure 3:**
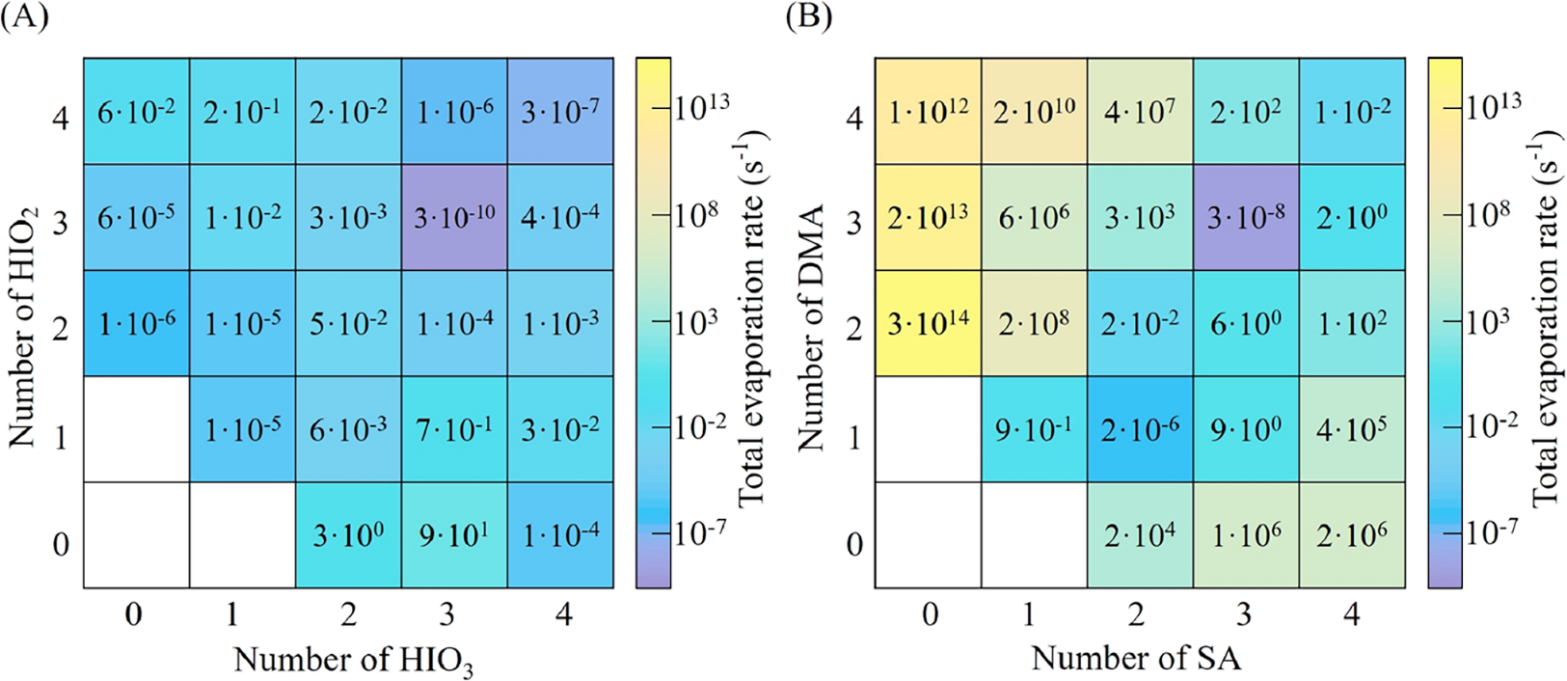
Evaporation rates of the (A) (HIO_3_)_*m*_(HIO_2_)_*n*_ and (B) (SA)_*m*_(DMA)_*n*_ (original data adopted from Xie et al.^60^) clusters (*m* = 0–4, *n* = 0–4) at 263.15 K and 1 atm.

The stability of clusters can be evaluated by their evaporation rates, and the difference between evaporation and collision rates (which are determined by ambient vapor concentrations) will determine whether a cluster shrinks or grows. Generally, the slower the evaporation rate is, the greater the cluster stability is.^24,60,61^ As shown in Figure 3A, all clusters except (HIO_3_)_2_ and (HIO_3_)_3_, have an evaporation rate below 1 s^–1^ at 263.15 K. Notably, more than half of the clusters have evaporation rates on the order of 10^–3^–10^–10^ s^–1^, indicating their high stability. The iodine oxoacid system has a larger number of stable clusters within a 4 × 4 box compared with the widely studied SA/MSA–base systems.^60,62^ The stable clusters consist of two types: (1) homomolecular clusters, i.e., (HIO_2_)_2_, (HIO_2_)_3_, and (HIO_3_)_4_, and (2) heteromolecular clusters, i.e., (HIO_3_)_1_(HIO_2_)_1_, (HIO_3_)_1_(HIO_2_)_2_, (HIO_3_)_2_(HIO_2_)_1_, (HIO_3_)_2_(HIO_2_)_3_, (HIO_3_)_3_(HIO_2_)_2_, (HIO_3_)_3_(HIO_2_)_3_, (HIO_3_)_3_(HIO_2_)_4_, (HIO_3_)_4_(HIO_2_)_3_, and (HIO_3_)_4_(HIO_2_)_4_, which lie on the diagonal line or its adjacent sites, and (HIO_3_)_4_(HIO_2_)_2_, which lies far from the diagonal line. Therefore, the location of stable clusters within a 4 × 4 box for the iodine oxoacid system differs from that of the SA/MSA–base systems, where stable clusters lie on or closely below the diagonal line.^24,60−62^ The difference in the distribution of stable clusters between the iodine oxoacid system and MSA/SA–base systems mainly results from the difference in the binding ability of the “base” molecules. HIO_2_ has strong binding with itself and with HIO_3_, while other atmospheric bases have weak binding with themselves and only have strong binding with acids. The greater number of stable clusters and their unique distribution provide the iodine oxoacid system with a much more flexible pathway for cluster growth than the SA/MSA–base systems (see the Cluster Growth Pathway section). In addition, the evaporation rates of all clusters above the diagonal line are significantly lower than those of the corresponding clusters below the diagonal line (except clusters (HIO_2_)_4_, (HIO_3_)_1_(HIO_2_)_4_, (HIO_3_)_2_(HIO_2_)_4_, and (HIO_3_)_2_(HIO_2_)_3_), implying that HIO_2_-rich paths can compete for nucleation despite their lower vapor concentrations. We note that a recent study employed master equation methods to calculate the collision rate coefficient and evaporation rate for the formation of (HIO_3_)_2_ and (HIO_3_)_1_(HIO_2_)_1_ dimers.^36^ Their collision rate coefficients are lower and their evaporation rates are higher than the values provided in this study (Table S6).

### Cluster Formation Rate

**Figure 4:**
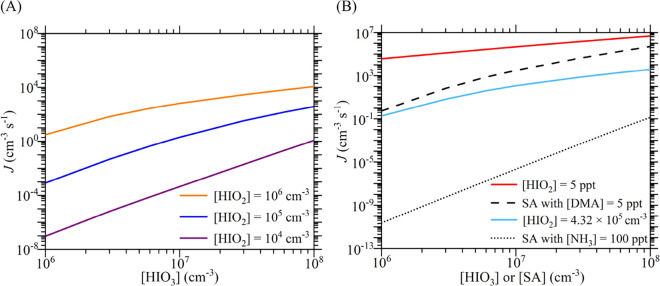
Comparison of neutral iodine oxoacid cluster formation rates (*J*) with neutral SA–NH_3_/DMA cluster formation rates at 263.15 K and CS = 2 × 10^–3^ s^–1^. (A) Iodine oxoacid cluster formation rates versus [HIO_3_] and [HIO_2_] and (B) comparison of iodine oxoacid cluster formation rates with SA–NH_3_/DMA cluster formation rates. The SA–DMA rates and SA–NH_3_ rates are calculated based on Δ*G* values from the DLPNO-CCSD(T)/aug-cc-pVTZ//ωB97X-D/6-31++G(d,p) level (also applying quasi-harmonic correction).^60^ The curves in panel (A) follow a power law, *J* ∝ [HIO_3_]^*n*^, with fitted slopes *n* of 1.7 ± 0.15 ([HIO_2_] = 10^6^ cm^–3^), 2.8 ± 0.15 ([HIO_2_] = 10^5^ cm^–3^), and 3.5 ± 0.04 ([HIO_2_] = 10^4^ cm^–3^).

Our calculated cluster formation rates (*J*) for iodine oxoacids at atmospheric [HIO_3_] and [HIO_2_] are presented in Figure 4A. *J* increases steeply with [HIO_3_] and [HIO_2_]; an increase of either [HIO_3_] or [HIO_2_] by one order of magnitude, while keeping the other constant, leads to an increase of 18–8786 times in *J*. To underscore the fast cluster formation rates of iodine oxoacids, we show in Figure 4B comparable theoretical calculations of the nucleation rates of SA–DMA and SA–NH_3_ at the same temperature (263.15 K) and CS (2 × 10^–3^ s^–1^). In Figure 4B, we determine the iodine oxoacid cluster formation rates under two conditions. In condition 1 (blue curve), all precursor concentrations correspond to their ambient range, thus showing the difference in their cluster formation ability in the atmosphere. In condition 2 (red curve), [HIO_3_] is set equal to [SA] and [HIO_2_] is set equal to [DMA], thus showing the difference in their intrinsic cluster formation ability (mainly determined by cluster formation free energies). As seen in Figure 4B, *J* for SA–DMA is higher than that for iodine oxoacids under condition 1, indicating that the overall cluster formation ability of SA–DMA is stronger than that of iodine oxoacids under ambient conditions. Under condition 2, *J* for SA–DMA is lower than that for iodine oxoacids, especially under low [SA] or [HIO_3_], indicating the lower intrinsic cluster formation ability of SA–DMA compared with that of iodine oxoacids. This comparison shows that the availability of either HIO_2_ or DMA is the main determinant of whether iodine oxoacids or SA–DMA, respectively, is the faster nucleation mechanism. Moreover, since both HIO_3_ and HIO_2_ originate from the same iodine sources, there is a high probability that, when one is present, both are present, which favors iodine oxoacid nucleation. Additionally, the *J* value for iodine oxoacids at [HIO_2_] = 4.32 × 10^5^ cm^–3^ is much faster than that for SA–NH_3_ at [NH_3_] = 100 ppt (about 2.79 × 10^9^ cm^–3^ at 263.15 K) for [HIO_3_] and [SA] over the range 10^6^ to 10^8^ cm^–3^. Therefore, the cluster formation capability of iodine oxoacids is expected always to be larger than that of SA with 100 ppt NH_3_, which is consistent with CLOUD measurements.^35^ In addition, it was found that the selection of CS value does not significantly change the revealed trend for the formation rates of these three systems by test simulations with CS = 2 × 10^–2^ s^–1^ and 2 × 10^–4^ s^–1^ (Figure S2).

### Cluster Growth Pathway

**Figure 5:**
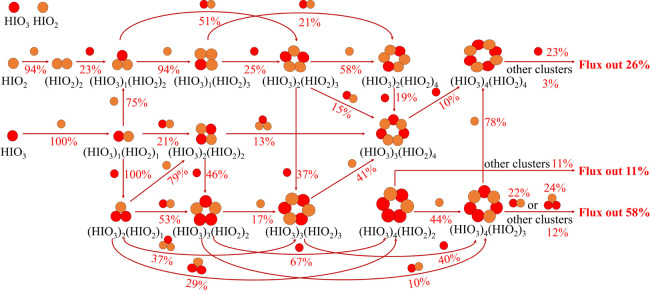
Neutral iodine oxoacid cluster growth pathways at *T* = 263.15 K with [HIO_3_] = 1.42 × 10^7^ cm^–3^, [HIO_2_] = 4.32 × 10^5^ cm^–3^, and CS = 2 × 10^–3^ s^–1^. The dark red lines give the dominant growth paths between clusters, the arrows indicate the direction of the flux, and the numbers represent the contribution percentage of a small cluster to a larger cluster along the direction of the arrow. The pathways contributing less than 10% to the flux of the cluster are not shown for clarity.

Figure 5 shows iodine oxoacid cluster growth pathways at 263.15 K (−10 °C) with [HIO_3_] = 1.42 × 10^7^ cm^–3^, [HIO_2_] = 4.32 × 10^5^ cm^–3^ (under the same concentrations as an experiment from CLOUD (Table S2)), and CS = 2 × 10^–3^ s^–1^. The cluster growth pathway is mainly driven by heteromolecular collisions involving HIO_3_ and HIO_2_, with a minor channel of homomolecular HIO_2_ collisions. Homomolecular HIO_3_ collisions have a negligible contribution to neutral iodine oxoacid cluster growth during nucleation, consistent with CLOUD results.^35^ After dimer formation, the major growth pathway becomes complicated. The (HIO_3_)_1_(HIO_2_)_1_ dimer collides with HIO_2_ or HIO_3_ to form the (HIO_3_)_1_(HIO_2_)_2_ or (HIO_3_)_2_(HIO_2_)_1_ trimer. Growth from the heteromolecular trimers continues via various pathways, eventually producing (HIO_3_)_4_(HIO_2_)_2_, (HIO_3_)_4_(HIO_2_)_3_, and (HIO_3_)_4_(HIO_2_)_4_ clusters. It deserves mentioning that the collision with small-sized clusters, i.e., (HIO_3_)_1_(HIO_2_)_1_, (HIO_3_)_2_(HIO_2_)_1_, or (HIO_3_)_1_(HIO_2_)_2_, is involved in the cluster growth besides collision with monomers. Previous studies also found that small clusters accelerate cluster growth in some cases of the SA–base, MSA–base, and HIO_3_–NH_3_ systems.^41,61,62^ In most of the previous studies,^24,60−62^ only coagulations of clusters on the diagonal line contribute to cluster growth, with smaller contributions of clusters immediately below the diagonal line. Surprisingly, it is found that three clusters far from the diagonal line ((HIO_3_)_4_(HIO_2_)_2_, (HIO_3_)_2_(HIO_2_)_4_, and (HIO_3_)_1_(HIO_2_)_3_) also contribute to cluster growth. The (HIO_3_)_4_(HIO_2_)_2_ cluster can directly collide with clusters to grow out of the 4 × 4 box, which accounts for 11% of the cluster formation rate. Alternatively, the (HIO_3_)_4_(HIO_2_)_2_ cluster can collide with HIO_2_ to form (HIO_3_)_4_(HIO_2_)_3_, which in turn has two growth pathways: the first is the collision with clusters such as (HIO_3_)_1_(HIO_2_)_1_ and (HIO_3_)_2_(HIO_2_)_1_, and the other is sequential addition of HIO_2_ and then HIO_3_ to finally form the (HIO_3_)_5_(HIO_2_)_4_ cluster, to grow out of the 4 × 4 box. The primary growth pathways emerging from (HIO_3_)_4_(HIO_2_)_2–4_ clusters contribute at least 95% to the overall cluster formation rate. The above cluster growth features for the iodine oxoacid system differ significantly from SA/MSA–base cluster formation mechanisms, which follow a more restricted stoichiometric path.^24,60−62^ In addition, it was found that the selection of the temperature and concentration of precursors can affect the growth pathways, while the growth was still dominated by mixed HIO_3_–HIO_2_ clusters by test simulation at different temperatures and concentrations of precursors (Figures S3–S5).

### Comparison with the CLOUD Experiment

**Figure 6:**
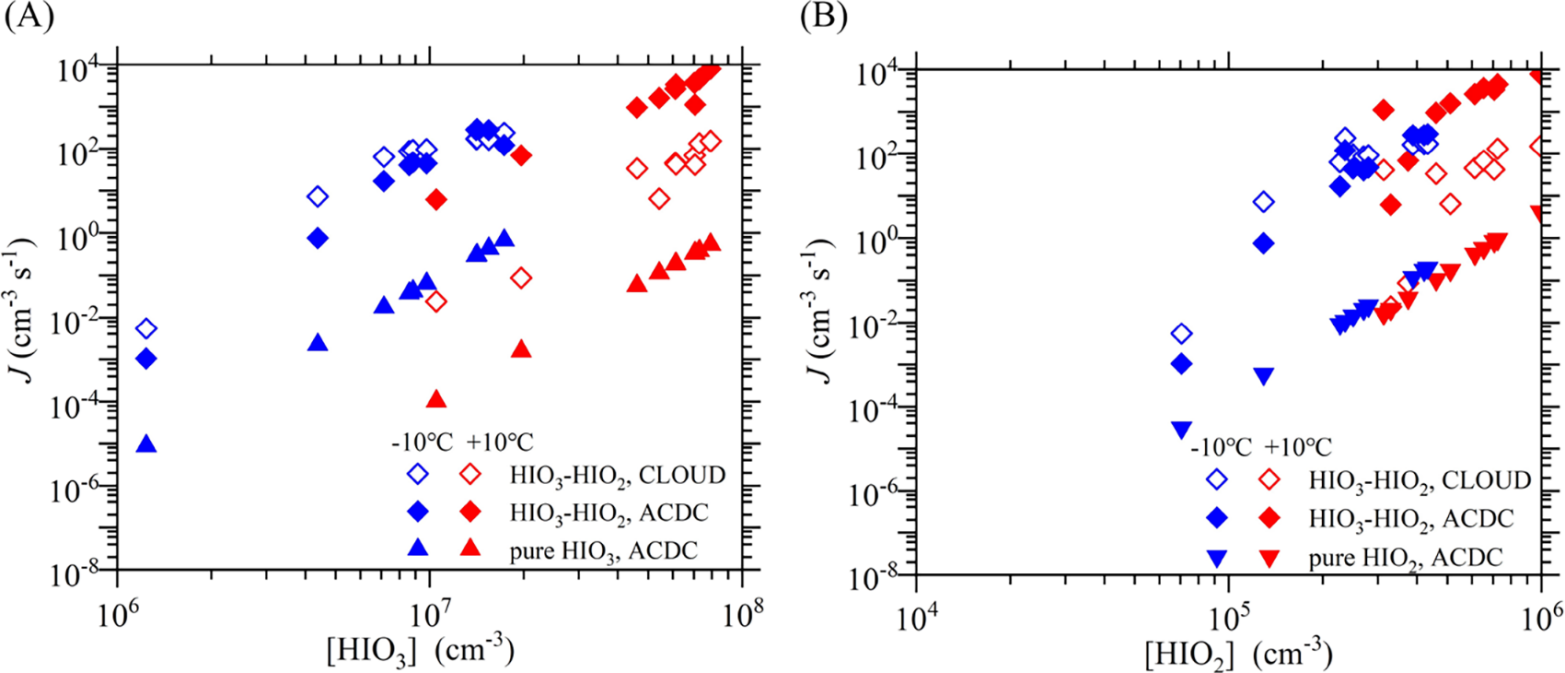
Measured (CLOUD) and simulated (ACDC) neutral cluster formation rates *J* versus (A) [HIO_3_] and (B) [HIO_2_] at +10 °C (red symbols) and −10 °C (blue symbols). The hollow diamonds show iodine oxoacid cluster formation rates from CLOUD. The filled symbols show cluster formation rates from ACDC simulations based on our quantum chemical calculations: iodine oxoacid clusters (filled diamonds), pure HIO_3_ clusters (filled pyramids), and pure HIO_2_ clusters (filled inverted pyramids).

**Figure 7:**
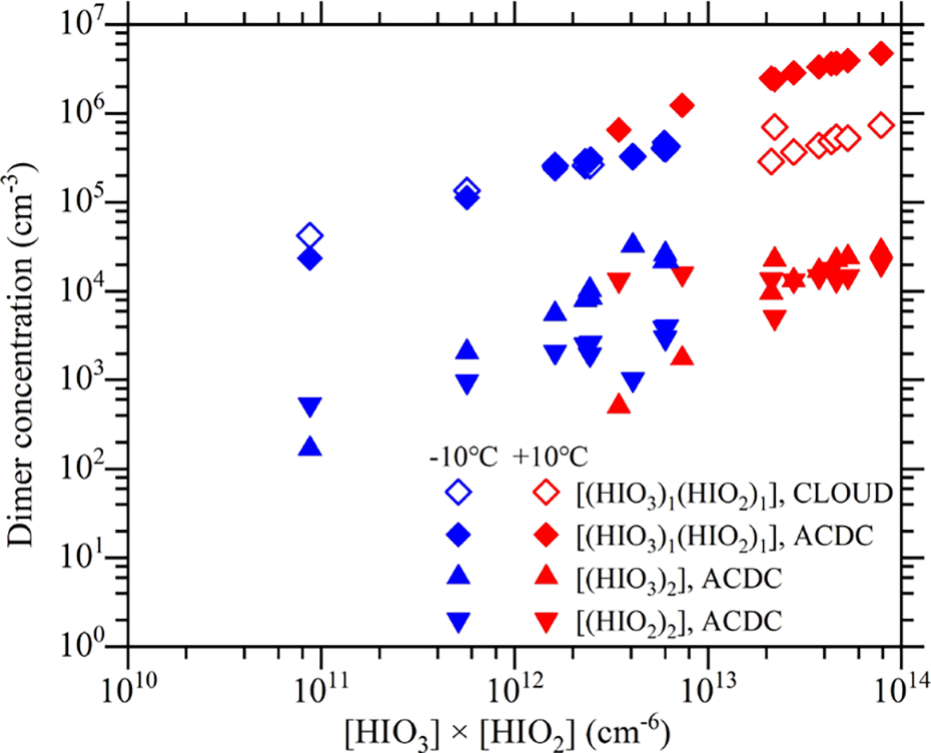
Measured (CLOUD) and simulated (ACDC) dimer concentrations ([(HIO_3_)_1_(HIO_2_)_1_], [(HIO_3_)_2_], and [(HIO_2_)_2_]) versus [HIO_3_] × [HIO_2_] (cm^–6^) at +10 °C (red symbols) and −10 °C (blue symbols). The hollow diamonds show [(HIO_3_)_1_(HIO_2_)_1_] measured by CLOUD. The filled symbols show dimer concentrations from ACDC simulations based on our quantum chemical calculations: filled diamonds for [(HIO_3_)_1_(HIO_2_)_1_], filled pyramids for [(HIO_3_)_2_], and filled inverted pyramids for [(HIO_2_)_2_].

To validate the predicted cluster concentrations and formation rates, here we compare our results with CLOUD measurements. The cluster formation rates at 1.7 nm of iodine oxoacids (HIO_2–3_) have been presented in a recent CLOUD study.^35^ Additionally, the (HIO_3_)_1_(HIO_2_)_1_ dimer concentration ([(HIO_3_)_1_(HIO_2_)_1_]) from the same set of experiments are reported here (Table S2). To compare with the experimental data, we have calculated *J* and [(HIO_3_)_1_(HIO_2_)_1_] under the same conditions as the CLOUD experiments, including precursor concentrations, wall loss rates for individual clusters, and temperatures. The iodine oxoacid cluster formation rates at +10 and −10 °C, from both the CLOUD study and this study are shown in Figure 6. For comparison, we also show in Figure 6 the simulated *J* for pure HIO_3_ clusters ((HIO_3_)_1–4_) and pure HIO_2_ clusters ((HIO_2_)_1–4_). In Figure 7, we show the calculated concentrations of homomolecular dimers ((HIO_3_)_2_ and (HIO_2_)_2_) and the heteromolecular dimer ((HIO_3_)_1_(HIO_2_)_1_) together with the [(HIO_3_)_1_(HIO_2_)_1_] measured by CLOUD.

As seen in Figure 6, the calculated *J* for iodine oxoacid clusters are one to four orders of magnitude faster than those for pure HIO_3_ and pure HIO_2_ clusters. This indicates that HIO_3_–HIO_2_ neutral cluster formation will dominate over HIO_3_–HIO_3_ and HIO_2_–HIO_2_ neutral cluster formation, as found experimentally by He et al.^35^ The same conclusion can be drawn from the high [(HIO_3_)_1_(HIO_2_)_1_] seen in Figure 7; the concentrations of (HIO_3_)_1_(HIO_2_)_1_ dimers are one to three orders of magnitude higher than those of (HIO_3_)_2_ or (HIO_2_)_2_ dimers. At −10 °C, the ratio of the calculated *J* divided by the measured *J* has a median value of 0.49 (Figure S6), a good agreement considering the systematic errors in both the experiments and our calculations (see below). Additionally, the calculated [(HIO_3_)_1_(HIO_2_)_1_] are in good agreement with the CLOUD experiments at −10 °C, with a median ratio of 1.07 (the ratio of the calculated [(HIO_3_)_1_(HIO_2_)_1_] divided by the measured value) (Figure S7). The good agreement on [(HIO_3_)_1_(HIO_2_)_1_] also suggests that (HIO_3_)_1_(HIO_2_)_1_ is well captured by the nitrate-CIMS and it is unlikely a surrogate for other iodine species as suggested by a recent study.^37^ Although the agreement between our calculations and the CLOUD data is good at −10 °C, the agreement is poorer at +10 °C, but nevertheless acceptable within theoretical and experimental uncertainties (see below). The median *J* ratio (ACDC/CLOUD) is 56 (Figure S6) but reaches as high as 789. The calculated [(HIO_3_)_1_(HIO_2_)_1_] at +10 °C are also higher than the measured values, with the highest ratio of 8.6 (Figure S7).

The poorer prediction for [(HIO_3_)_1_(HIO_2_)_1_] and *J* at +10 °C implies that the temperature dependency of the nucleation rate may not be well presented in ACDC. To assess this hypothesis, we examine the prediction of ACDC against SA–NH_3_ nucleation rates obtained from CLOUD at different temperatures.^63^ We find that predicted nucleation rates present much better consistency with the experimental results from CLOUD at −10, −30, and −50 °C than they do at +10 °C (Figure S8). This suggests that the temperature dependencies of both the iodine oxoacid and SA–NH_3_ systems are not accurately represented at present in ACDC, especially at +10 °C and potentially also in warmer conditions. Therefore, the temperature dependency of the quantum chemical calculations + ACDC methods and their comparison with experimental results warrant further study in the future. It is worth noting that despite the seemingly large difference factors at +10 °C, the agreement between our calculations and CLOUD is considered reasonable considering the *J* uncertainty of at least one order of magnitude in our simulations and a comparable uncertainty in CLOUD due to measurement uncertainties on vapor and particle concentrations. Moreover, when compared with those from previous studies,^14,40,42^ the nucleation rates calculated here show significantly improved agreement with the CLOUD experimental data.

## Implications

Our study reveals that the mixture of HIO_3_ and HIO_2_ vapors has an extremely high potential to form molecular clusters. We find that the iodine oxoacid system is even more (intrinsically) efficient for particle nucleation than the SA–DMA system, which is known to introduce rapid nucleation in urban environments.^53^ Owing to the lower concentrations of HIO_2_, the overall nucleation rates observed from the iodine oxoacid system in pristine atmospheres are lower than that of SA–DMA observed in the polluted boundary layer. However, in pristine marine areas where base vapors are scarce, iodine oxoacid nucleation may provide the dominant source of new particles. Furthermore, since both HIO_3_ and HIO_2_ derive from the same precursor vapors, they are naturally found together, in contrast with SA and DMA/NH_3_, which are emitted by unrelated sources. That makes iodine oxoacid nucleation an especially efficient source of new particles in pristine areas.

Our study reveals the unexpected base behavior of HIO_2_ (accepting a proton from HIO_3_) and the stronger halogen bonding of HIO_2_ compared with that of HIO_3_. These characteristics produce highly stable HIO_3_–HIO_2_ clusters. The base behavior and strong halogen bonding of HIO_2_ suggest that it may potentially be able to stabilize other organic and inorganic acids to form particles. Combined with the fact that iodine levels have tripled since the 1950s because of the anthropogenic ozone increases and thinning sea ice,^64−66^ iodine oxoacid nucleation could become more important than what we thought, especially in the regions of the atmosphere where iodine oxoacids and organic and inorganic acids can coexist and base vapors such as ammonia and amines are scarce. This warrants further studies on a potentially wider role of iodine oxoacids in aerosol nucleation of marine atmospheres. In addition, this study provides necessary thermodynamic data for the three branches of neutral iodine oxoacid cluster formation, which can be parameterized for simulating iodine oxoacid particle formation in the climate models in the future.

## Supporting Information Available

Details of the physical principles of ACDC; selection of boundary clusters; estimation of the enhancing factor for the collision of iodine oxoacid systems; effects of mixing basis sets of different sizes for calculating the Δ*G* values of (HIO_3_)_0–3_(HIO_2_)_0–3_ clusters; measured concentrations of HIO_3_, HIO_2_, and (HIO_3_)_1_(HIO_2_)_1_ in the CLOUD experiments; wall loss rates for individual clusters deduced from CLOUD experiments; geometries, Δ*G* values, collision rate coefficients, and evaporation rates of dimers from previous works; bond length and energy gap between BD* and LP for halogen bonds formed in (HIO_3_)_2_ and (HIO_2_)_2_; Δ*G* with quasi-harmonic correction of (HIO_3_)_*m*_(HIO_2_)_*n*_ clusters (*m* = 0–4, *n* = 0–4) at 298.15 K; effects of the CS value for main results; cluster growth pathways at 283.15 K and various precursor concentrations; difference factors, *R* and *r*, between CLOUD measurements and ACDC prediction; measured (CLOUD) and simulated (ACDC) cluster formation rates for the SA–NH_3_ system (PDF)
