# Supplementary material for: Critical Role of Iodous Acid in Neutral Iodine Oxoacid Nucleation
Source: Environ Sci Technol. 2022 Sep 20;56(19):14166–77. doi: 10.1021/acs.est.2c04328 (PMC9536010; doi:10.1021/acs.est.2c04328)
Supplement: Supplementary file 1 — es2c04328_si_001.pdf [file es2c04328_si_001.pdf]

Supporting Information

Critical Role of Iodous Acid in Neutral Iodine Oxoacid Nucleation

Rongjie Zhang[1], Hong-Bin Xie[1]*, Fangfang Ma[1], Jingwen Chen[1], Siddharth Iyer[2], Mario Simon[3], Martin Heinritzi[3], Jiali Shen[4], Yee Jun Tham[5], Theo Kurtén[6], Douglas R. Worsnop[4,7], Jasper Kirkby[8,3], Joachim Curtius[3], Mikko Sipilä[4], Markku Kulmala[4,9,10] and Xu-Cheng He[4,11]*

[1]Key Laboratory of Industrial Ecology and Environmental Engineering (Ministry of Education), School of Environmental Science and Technology, Dalian University of Technology, Dalian 116024, China

[2]Aerosol Physics Laboratory, Faculty of Engineering and Natural Sciences, Tampere University, Tampere 33014, Finland

[3]Institute for Atmospheric and Environmental Sciences, Goethe University Frankfurt, Frankfurt am Main 60438, Germany

[4]Institute for Atmospheric and Earth System Research/Physics, Faculty of science, University of Helsinki, Helsinki 00014, Finland

[5]School of Marine Sciences, Sun Yat-sen University, Zhuhai 519082, China

[6]Department of Chemistry, University of Helsinki, Helsinki 00014, Finland

[7]Aerodyne Research, Inc., Billerica, Massachusetts 01821, United States

[8]CERN, the European Organization for Nuclear Research, CH-1211 Geneva, Switzerland

[9]Joint International Research Laboratory of Atmospheric and Earth System Sciences, School of Atmospheric Sciences, Nanjing University, Nanjing 210023, China

Supporting Information

[10]Aerosol and Haze Laboratory, Beijing Advanced Innovation Center for Soft Matter Science and Engineering, Beijing University of Chemical Technology, Beijing 100029, China

[11]Center for Atmospheric Particle Studies, Carnegie Mellon University, Pittsburgh, Pennsylvania 15213, United States

In total, 45 pages, 8 figures and 6 tables

Supporting Information

Contents

1. Physical Principles of ACDC S4
2. Selection of Boundary Clusters S4
3. Estimation of enhancing factor for collision of iodine oxoacids system S5
4. Table S1 .... S6
5. Table S2 .... S7
6. Table S3 .... S8
7. Table S4 .... S9
8. Table S5 .... S10
9. Table S6 .... S11
10. Figure S1 .... S12
11. Figure S2 .... S12
12. Figure S3 .... S13
13. Figure S4 .... S14
14. Figure S5 .... S15
15. Figure S6 .... S16
16. Figure S7 .... S17
17. Figure S8 .... S18
18. Coordinates of all optimized $(HIO_3)_m(HIO_2)_n$ (m = 0-4, n = 0-4) clusters . S19
19. References S42

Supporting Information

Physical Principles of ACDC. Briefly, the core of ACDC is to employ the birth-death equation (Eq. (1)) to describe the time-dependent cluster distributions:

$$\frac{dc_i}{dt} = \frac{1}{2}\sum_{j<i}\beta_{j,(i\text{-}j)}c_j c_{(i\text{-}j)} + \sum_{j}\gamma_{(i+j)\rightarrow i}c_{i+j} - \sum_{j}\beta_{i,j}c_i c_j - \frac{1}{2}\sum_{j<i}\gamma_{i\rightarrow j}c_i + Q_i - S_i \quad (1)$$

where subscripts (i, j, i-j, j-i and i+j) represent different clusters or monomers in the system, c_i represents the number concentration of i, $\beta_{i,j}$ denotes the collision rate coefficient between i and j, $\gamma_{(i+j)\rightarrow i}$ denotes the evaporation rate of a cluster i+j into smaller clusters (or monomer) i and j. Q_i represents an additional outside source term of i and S_i represents other sink terms for i. The collision rate coefficients were calculated by hard sphere kinetic gas theory as:

$$\beta_{i,j} = \left(\frac{3}{4\pi}\right)^{\frac{1}{6}}\left(\frac{6k_bT}{m_i} + \frac{6k_bT}{m_j}\right)^{\frac{1}{2}}\left(V_i^{\frac{1}{3}} + V_j^{\frac{1}{3}}\right)^2 \quad (2)$$

where k_b is the Boltzmann constant, T is the temperature, and m_i and V_i are the mass and volume of i, respectively. The evaporation rates were calculated using detailed balance as:

$$\gamma_{(i+j)\rightarrow i} = \beta_{i,j}c_{\mathrm{ref}}\exp\left\{\frac{\Delta G_{i+j} - \Delta G_i - \Delta G_j}{k_bT}\right\} \quad (3)$$

where ΔG is the formation free energy of the cluster, c_{ref} is the reference monomer concentration at 1 atm, which is the pressure at which ΔG was calculated.

Selection of Boundary Clusters. In ACDC simulation, the boundary clusters are ones allowed to flux out the simulation box for further growth, therefore, these clusters are required to have favorable compositions for high stability (low evaporation rate).[1] In the studied iodine oxoacid system, most of $(HIO_3)_z(HIO_2)_z$ (z = 3-4), $(HIO_3)_{z+1}(HIO_2)_z$

(z = 1-3) and $(HIO_3)_z(HIO_2)_{z+1}$ (z = 1-3) clusters have relatively low evaporation rates. For example, the larger $(HIO_3)_z(HIO_2)_z$, $(HIO_3)_{z+1}(HIO_2)_z$ and $(HIO_3)_z(HIO_2)_{z+1}$ clusters have evaporation rate less than 10^{-3} s^{-1} and therefore can be deemed as stable clusters. This allows us to select $(HIO_3)_5(HIO_2)_4$ and $(HIO_3)_4(HIO_2)_5$ as the boundary clusters for iodine oxoacid system in the 4 × 4 box simulation. For pure HIO_3 system of $(HIO_3)_{1-4}$ clusters and pure HIO_2 system of $(HIO_2)_{1-4}$ clusters, $(HIO_3)_5$ and $(HIO_2)_5$ clusters are assumed to have low evaporation rates to leave their simulation box for further growth.

Estimation of Enhancing Factor for Collision of Iodine Oxoacids System. We calculated the polarizability of HIO_3 and HIO_2. The enhancing factor of 1.42 for the collision rate between HIO_3 and HIO_2 molecules is obtained by calculating the ratio of dispersion interaction (explicitly considering polarizability) induced collision rate[2] to one calculated by hard sphere kinetic gas theory. The corresponding enhancing factor for two SA molecules was calculated to be 1.35. Therefore, the enhancing factor for HIO_3-HIO_2 is about 1.05 times higher than that of SA-SA system when dispersion interaction induced collision rate was used. When the dipole-dipole induced collision rate[2] was used, the enhancing factor for HIO_3-HIO_2 (1.64) is also about 1.05 times higher than that of SA-SA system (1.55). Since reported the enhancing factor for two SA molecules is 2.3,[3,4] higher than our calculated values based on dispersion interaction (1.35) or dipole-dipole interaction (1.55), we estimated the enhancing factor for HIO_3-HIO_2 system by 2.3 multiplied by 1.05. The calculated factor is 2.4.

Supporting Information

Table S1. Comparison of ΔG (kcal mol^{-1}) for $(HIO_3)_{0\text{-}3}(HIO_2)_{0\text{-}3}$ clusters calculated at DLPNO-CCSD(T)/Basis2//M06-2X/Basis1 and DLPNO-CCSD(T)/Basis2//M06-2X/Basis2 level of theory at 298.15 K and 1 atm (Basis1 represents 6-31++G(d,p) for H, O atoms and aug-cc-pVTZ-PP with ECP28 for I atom and Basis2 represents aug-cc-pVTZ for H, O atoms and aug-cc-pVTZ-PP with ECP28 for I atom).

Clusters	ΔG (DLPNO-CCSD(T)/Basis2 //M06-2X/Basis1)	ΔG (DLPNO-CCSD(T)/Basis2 //M06-2X/Basis2)	Error
$(HIO_3)_1(HIO_2)_1$	-16.65	-16.52	0.13
$(HIO_3)_2$	-9.73	-9.46	0.27
$(HIO_2)_2$	-17.69	-17.42	0.27
$(HIO_3)_1(HIO_2)_2$	-34.39	-34.45	-0.06
$(HIO_3)_2(HIO_2)_1$	-30.02	-29.93	0.09
$(HIO_3)_3$	-18.20	-18.03	0.17
$(HIO_2)_3$	-33.69	-33.49	0.20
$(HIO_3)_2(HIO_2)_2$	-46.72	-46.90	-0.18
$(HIO_3)_3(HIO_2)_1$	-40.92	-41.29	-0.37
$(HIO_3)_1(HIO_2)_3$	-47.66	-47.68	-0.02
$(HIO_3)_3(HIO_2)_2$	-62.43	-62.65	-0.22
$(HIO_3)_2(HIO_2)_3$	-64.77	-64.86	-0.09
$(HIO_3)_3(HIO_2)_3$	-86.86	-87.05	-0.19

Table S2. Measured [HIO_3], [HIO_2], and [$(HIO_3)_1(HIO_2)_1$] (cm^{-3}) at +10 °C and -10 °C in CLOUD experiments.[5]

Temperature	[HIO_3]	[HIO_2]	[$(HIO_3)_1(HIO_2)_1$]
	1.05×10^{7}	3.30×10^{5}	-
	1.96×10^{7}	3.74×10^{5}	-
	4.60×10^{7}	4.60×10^{5}	2.88×10^{5}
	5.42×10^{7}	5.11×10^{5}	3.68×10^{5}
+10 °C	6.10×10^{7}	6.12×10^{5}	4.36×10^{5}
	6.13×10^{7}	7.06×10^{5}	4.86×10^{5}
	7.03×10^{7}	6.54×10^{5}	5.40×10^{5}
	7.06×10^{7}	3.13×10^{5}	7.02×10^{5}
	7.29×10^{7}	7.24×10^{5}	5.27×10^{5}
	7.91×10^{7}	9.92×10^{5}	7.35×10^{5}
	1.24×10^{6}	7.05×10^{4}	4.23×10^{4}
	4.38×10^{6}	1.29×10^{5}	1.36×10^{5}
	7.14×10^{6}	2.27×10^{5}	2.57×10^{5}
	8.59×10^{6}	2.70×10^{5}	2.59×10^{5}
-10 °C	8.83×10^{6}	2.80×10^{5}	2.64×10^{5}
	9.76×10^{6}	2.50×10^{5}	2.58×10^{5}
	1.41×10^{7}	4.22×10^{5}	4.06×10^{5}
	1.42×10^{7}	4.32×10^{5}	4.26×10^{5}
	1.55×10^{7}	3.88×10^{5}	4.01×10^{5}
	1.73×10^{7}	2.35×10^{5}	3.26×10^{5}

Table S3. Wall loss rates (s^{-1}) for $(HIO_3)_{0-4}(HIO_2)_{0-4}$ clusters at +10 ℃ and -10 ℃ deduced from CLOUD experiments.[5,6]

Species	Wall loss (+10 ℃)	Wall loss (-10 ℃)
HIO_3	2.36×10^{-3}	2.23×10^{-3}
HIO_2	2.41×10^{-3}	2.28×10^{-3}
$(HIO_3)_2$	2.03×10^{-3}	1.92×10^{-3}
$(HIO_3)_3$	1.85×10^{-3}	1.75×10^{-3}
$(HIO_3)_4$	1.73×10^{-3}	1.64×10^{-3}
$(HIO_2)_2$	2.07×10^{-3}	1.96×10^{-3}
$(HIO_2)_3$	1.89×10^{-3}	1.79×10^{-3}
$(HIO_2)_4$	1.77×10^{-3}	1.67×10^{-3}
$(HIO_3)_1(HIO_2)_1$	2.05×10^{-3}	1.94×10^{-3}
$(HIO_3)_1(HIO_2)_2$	1.88×10^{-3}	1.78×10^{-3}
$(HIO_3)_1(HIO_2)_3$	1.76×10^{-3}	1.66×10^{-3}
$(HIO_3)_1(HIO_2)_4$	1.67×10^{-3}	1.58×10^{-3}
$(HIO_3)_2(HIO_2)_1$	1.86×10^{-3}	1.76×10^{-3}
$(HIO_3)_2(HIO_2)_2$	1.75×10^{-3}	1.65×10^{-3}
$(HIO_3)_2(HIO_2)_3$	1.66×10^{-3}	1.57×10^{-3}
$(HIO_3)_2(HIO_2)_4$	1.59×10^{-3}	1.50×10^{-3}
$(HIO_3)_3(HIO_2)_1$	1.74×10^{-3}	1.64×10^{-3}
$(HIO_3)_3(HIO_2)_2$	1.65×10^{-3}	1.56×10^{-3}
$(HIO_3)_3(HIO_2)_3$	1.58×10^{-3}	1.50×10^{-3}
$(HIO_3)_3(HIO_2)_4$	1.53×10^{-3}	1.44×10^{-3}
$(HIO_3)_4(HIO_2)_1$	1.64×10^{-3}	1.56×10^{-3}
$(HIO_3)_4(HIO_2)_2$	1.58×10^{-3}	1.49×10^{-3}
$(HIO_3)_4(HIO_2)_3$	1.52×10^{-3}	1.44×10^{-3}
$(HIO_3)_4(HIO_2)_4$	1.47×10^{-3}	1.39×10^{-3}

Table S4. Recalculated formation free energies (Δ*G*, kcal mol^{-1}) with quasi-harmonic correction based on the reported geometries of $(HIO_3)_2$, $(HIO_2)_2$ and $(HIO_3)_1(HIO_2)_1$ dimers at the DLPNO-CCSD(T)/aug-cc-pVTZ-(PP)//M06-2X/6-31++G(d,p) + aug-cc-pVTZ-PP level. The calculations are performed at 263.15 K and 1atm.

Cluster	Structure	Δ*G*
$(HIO_3)_2$ (Khanniche et al., 2016)[7]	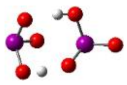	-10.91
$(HIO_3)_2$ (Zhang et al., 2020)[8]	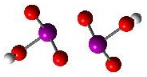	-11.01
$(HIO_3)_2$ (Kumar et al., 2018)[9]	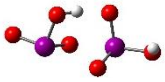	-11.42
$(HIO_3)_2$ (He et al., 2021)[5]	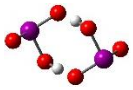	-9.42
$(HIO_2)_2$ (Zhang et al., 2022)[10]	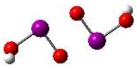	-19.24
$(HIO_3)_1(HIO_2)_1$ (Kumar et al., 2018)[9]	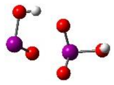	-14.24

Table S5. Bond length (Å) and energy gap (a.u.) between δ^* (O-I) and LP(O) for two types of halogen bond (XB) in the $(HIO_2)_2$ and $(HIO_3)_2$ clusters calculated at the M06-2X/6-31++G(d,p) + aug-cc-pVTZ-PP level of theory.

XB type	Bond length	Energy gap between δ^* (O-I) and LP(O)
O-I (from HIO_3)···O (from HIO_3)	2.34	0.36168
O-I (from HIO_2)···O (from HIO_2)	2.20	0.34007

Table S6. Collision rate coefficients (cm^3 s^{-1}) and evaporation rates (s^{-1}) for the formation of $(HIO_3)_2$ and $(HIO_3)_1(HIO_2)_1$ dimer calculated by master equation methods[11] and the hard sphere kinetic gas theory from ACDC.

Reaction	k (295 K) (master equation methods)	k (295 K) (hard sphere kinetic gas theory)
$HIO_3 + HIO_3 \rightarrow (HIO_3)_2$	1.1×10^{-11} cm^3 s^{-1}	6.0×10^{-10} cm^3 s^{-1}
$(HIO_3)_2 \rightarrow HIO_3 + HIO_3$	1388 s^{-1}	356 s^{-1}
$HIO_3 + HIO_2 \rightarrow (HIO_3)_1(HIO_2)_1$	6.7×10^{-11} cm^3 s^{-1}	6.5×10^{-10} cm^3 s^{-1}
$(HIO_3)_1(HIO_2)_1 \rightarrow HIO_3 + HIO_2$	0.8 s^{-1}	0.006 s^{-1}

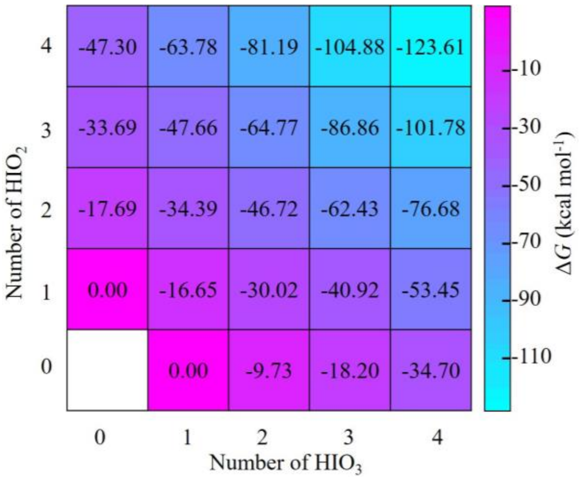

Figure S1. Formation free energy (ΔG) with quasi-harmonic correction of $(HIO_3)_m(HIO_2)_n$ clusters (m = 0-4, n = 0-4) calculated at the DLPNO-CCSD(T)/aug-cc-pVTZ-(PP)//M06-2X/6-31++G(d,p) + aug-cc-pVTZ-PP level. The calculations are performed at 298.15 K and 1 atm.

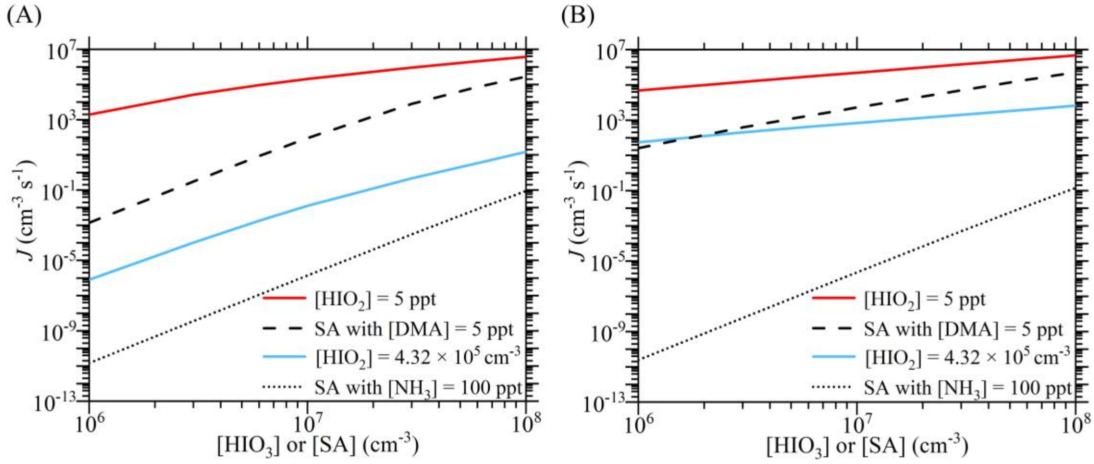

Figure S2. Comparison of neutral iodine oxoacid cluster formation rates (J) with neutral SA-NH_3/DMA cluster formation rates at 263.15 K and CS = 2 × 10^{-2} s^{-1} (A) or CS = 2 × 10^{-4} s^{-1} (B).

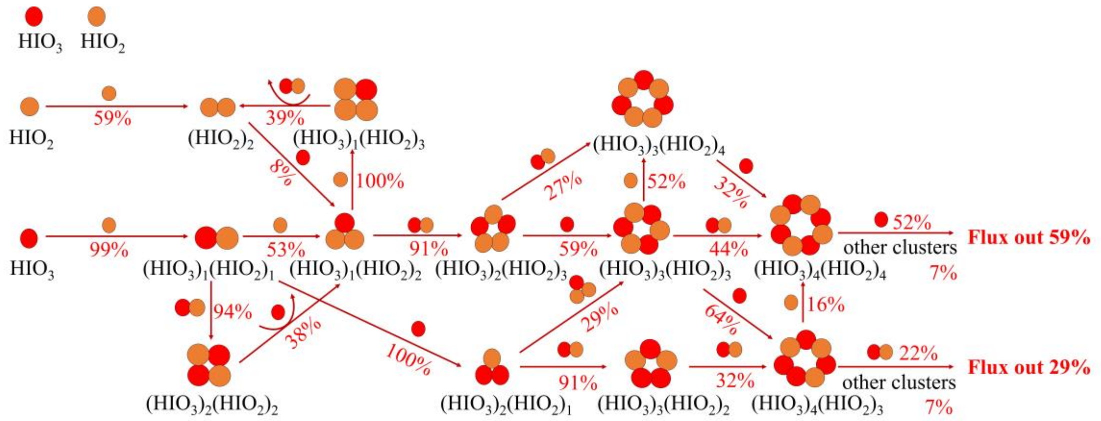

Figure S3. Neutral iodine oxoacid cluster growth pathways at T = 283.15 K with [HIO_3] = 1.42 × 10^7 cm^{-3}, [HIO_2] = 4.32 × 10^5 cm^{-3}, and CS = 2 × 10^{-3} s^{-1}. The dark red lines give the dominant growth paths between clusters, the arrows indicate the direction of the flux, and the numbers represent the contribution percentage of a small cluster to a larger cluster along the direction of the arrow. The pathways contributing less than 10% to the flux of the cluster are not shown except $(HIO_2)_2 \rightarrow (HIO_3)_1(HIO_2)_2$ for clarity.

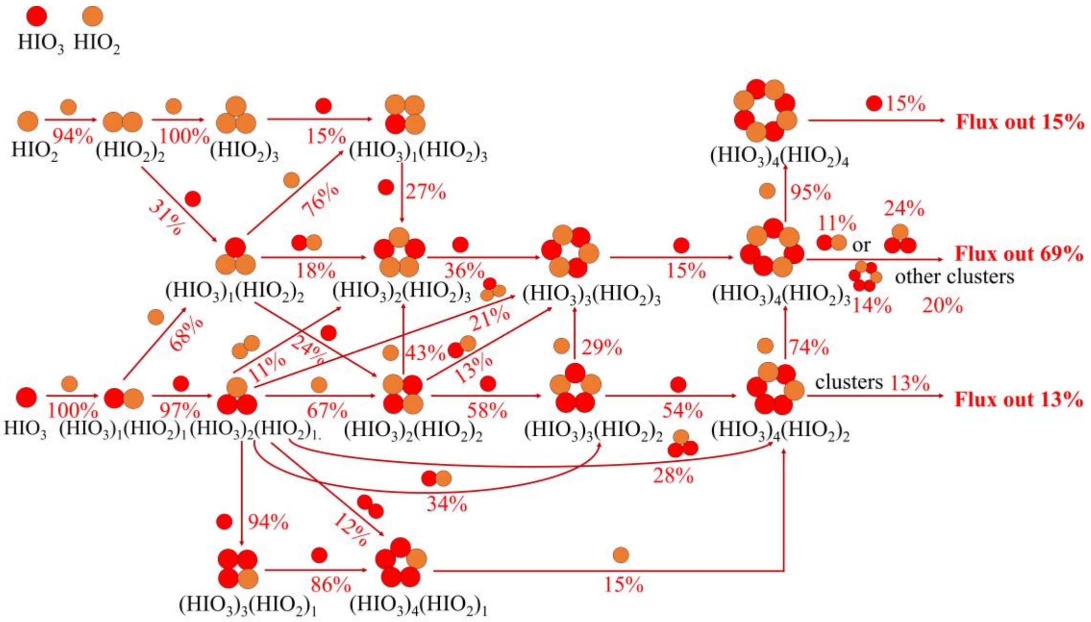

Figure S4. Neutral iodine oxoacid cluster growth pathways at T = 263.15 K with [HIO_3] = 1.42×10^8 cm^{-3}, [HIO_2] = 4.32×10^6 cm^{-3}, and CS = 2×10^{-3} s^{-1}. The dark red lines give the dominant growth paths between clusters, the arrows indicate the direction of the flux, and the numbers represent the contribution percentage of a small cluster to a larger cluster along the direction of the arrow. The pathways contributing less than 10% to the flux of the cluster are not shown for clarity.

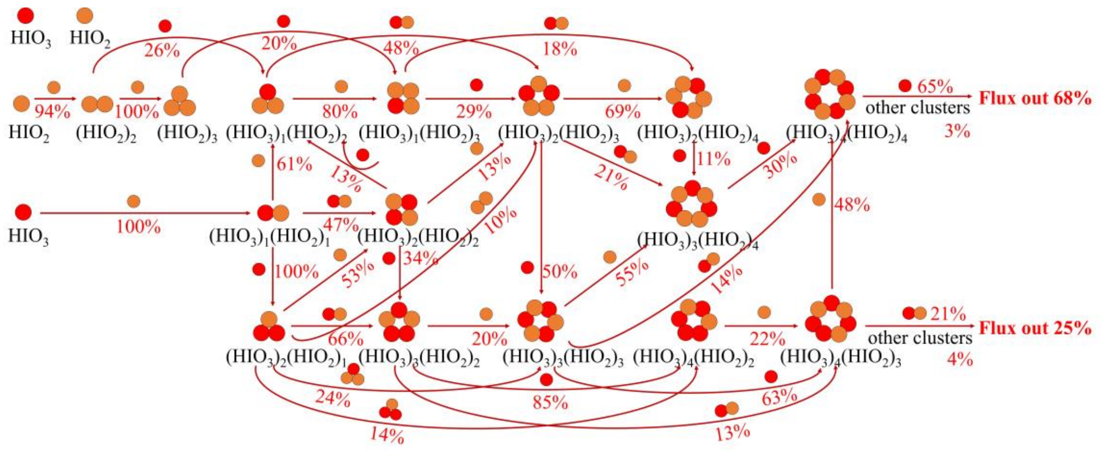

Figure S5. Neutral iodine oxoacid cluster growth pathways at T = 263.15 K with [HIO_3] = 1.42×10^{6} cm^{-3}, [HIO_2] = 4.32×10^{4} cm^{-3}, and CS = 2×10^{-3} s^{-1}. The dark red lines give the dominant growth paths between clusters, the arrows indicate the direction of the flux, and the numbers represent the contribution percentage of a small cluster to a larger cluster along the direction of the arrow. The pathways contributing less than 10% to the flux of the cluster are not shown for clarity.

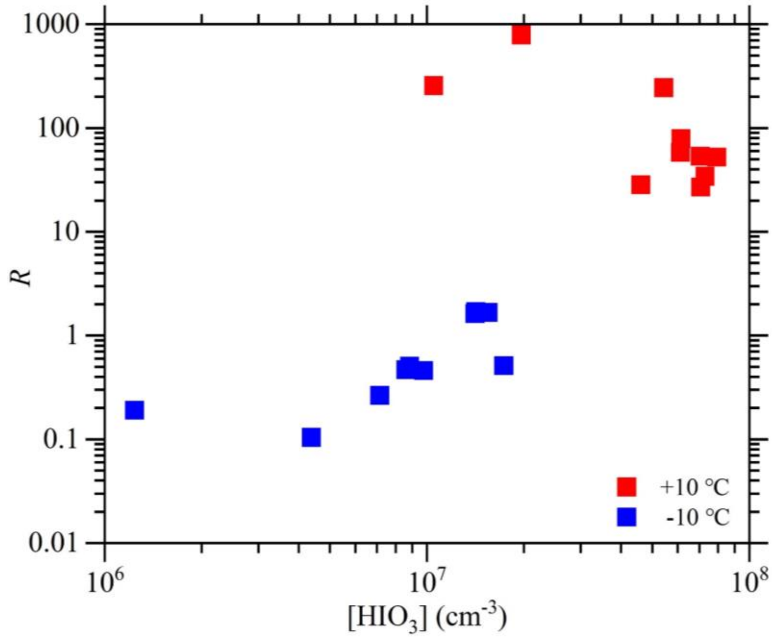

Figure S6. Variation of difference factor *R* (the ratio of calculated *J* divided by CLOUD measurements) against [HIO_3] at +10 ℃ and -10 ℃.

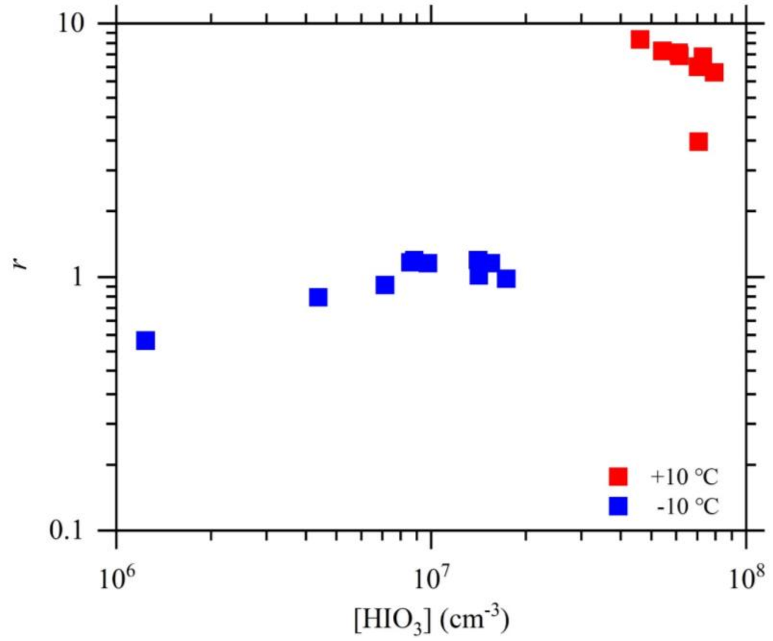

Figure S7. Variation of difference factor r (the ratio of calculated [$(HIO_3)_1(HIO_2)_1$] divided by CLOUD measurements) against [HIO_3] at +10 ℃ and -10 ℃.

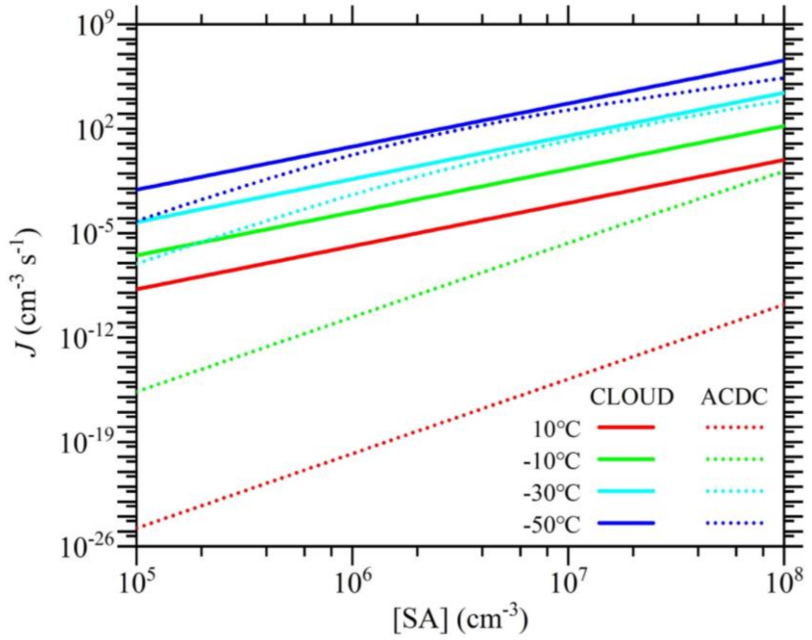

Figure S8. Measured (CLOUD) and simulated (ACDC) neutral SA-NH_3 (100 ppt) cluster formation rates versus [SA] at +10 °C, -10 °C, -30 °C and -50 °C. Measured rates are adopted from Dunne et al., 2016[12] and simulated rates are calculated based on ΔG values from DLPNO-CCSD(T)/aug-cc-pVTZ//ωB97X-D/6-31++G(d,p) level (also applying quasi-harmonic correction).[13]

Supporting Information

Coordinates of all optimized $(HIO_3)_m(HIO_2)_n$ (m = 0-4, n = 0-4) clusters.

HIO_3

I	-0.096607	0.000067	-0.244569
O	1.769042	-0.002142	0.230810
H	1.862877	-0.000985	1.199130
O	-0.679087	1.432383	0.619652
O	-0.682791	-1.430560	0.619917

HIO_2

O	-1.303796	1.080120	-0.013772
I	-0.088359	-0.274376	0.005178
O	1.656531	0.611776	-0.113825
H	1.861147	1.006760	0.746338

$(HIO_3)_2$

I	1.632311	-0.188706	-0.228750
O	3.410861	0.037330	0.468618
H	3.409781	0.638688	1.231177
O	0.822183	-0.559726	1.327671
O	1.251132	1.547796	-0.459091
H	-0.338124	1.787880	0.043563
I	-1.750037	-0.157202	0.211881
O	-1.297203	1.671133	0.329376

O	-3.274780	-0.079754	-0.664892
O	-0.516214	-0.628459	-1.049264

$(HIO_3)_3$

I	-0.041936	-1.962937	-0.237805
O	-1.302384	-3.316635	0.322136
H	-1.039341	-3.670649	1.187249
O	-0.318825	1.882209	-0.079527
O	1.346557	-2.643777	0.615605
I	-1.866302	0.955085	0.247817
O	-2.965555	2.431488	-0.344926
H	-2.914538	2.536449	-1.308147
O	-2.117799	-0.020300	-1.220862
O	-0.703521	-0.728635	0.972151
I	1.824010	1.199235	-0.096747
O	3.672173	0.708103	-0.345727
H	3.882597	-0.087729	0.171170
O	1.750168	0.857881	1.633358
O	1.206102	-0.285510	-0.983874

$(HIO_3)_4$

I	2.869524	-0.027027	-0.847525
O	3.327263	-0.611893	0.777839

O	1.885931	1.437060	-0.527763
O	4.519498	0.904423	-1.180028
H	4.799616	1.407414	-0.398005
I	0.138386	-2.002622	0.757733
O	-0.200456	-0.532082	1.703599
O	1.014170	-1.398565	-0.724666
O	1.705164	-2.575383	1.674094
H	2.428164	-1.912196	1.537272
I	-0.222455	2.017887	0.701174
O	-1.010942	1.055725	-0.672069
O	-0.124027	3.633609	0.019084
O	-1.807868	2.225627	1.720263
H	-2.483471	1.547892	1.450128
I	-2.750948	-0.217455	-0.907062
O	-3.420888	0.392473	0.629810
O	-1.788698	-1.685735	-0.416324
O	-4.325414	-1.263060	-1.307294
H	-4.763073	-1.552176	-0.490692

$(HIO_2)_2$

O	-3.188864	-0.790383	-0.105651
I	-1.574686	0.346471	0.005838

O	-0.457680	-1.196947	-0.014427
H	-3.266029	-1.338256	0.687342
O	0.457728	1.196997	0.016453
I	1.574660	-0.346454	-0.005966
O	3.189182	0.790027	0.104479
H	3.264489	1.339785	-0.687397

$(HIO_2)_3$

O	-2.925705	2.039857	-0.024116
I	-0.952462	1.820291	-0.150799
O	-0.987085	0.356749	1.049600
H	-3.174786	2.374233	0.847665
O	0.231047	-1.050584	-0.951542
I	-1.128064	-1.660825	0.217384
O	-0.955952	-3.511591	-0.476299
H	-1.220560	-3.551389	-1.405322
O	1.199949	1.507783	-0.251709
I	2.057948	-0.168062	-0.074868
O	3.694634	0.723630	0.595037
H	3.536853	1.106027	1.468847

$(HIO_2)_4$

I	0.563773	2.180723	0.805374

O	-0.251298	1.595606	-0.791320
O	-0.563773	3.823084	0.857613
H	-0.300124	4.433186	0.155981
I	-0.563773	-2.180723	0.805374
O	0.251298	-1.595606	-0.791320
O	0.563773	-3.823084	0.857613
H	0.300124	-4.433186	0.155981
I	-2.180723	0.563773	-0.805374
O	-1.595606	-0.251298	0.791320
O	-3.823084	-0.563773	-0.857613
H	-4.433186	-0.300124	-0.155981
I	2.180723	-0.563773	-0.805374
O	1.595606	0.251298	0.791320
O	3.823084	0.563773	-0.857613
H	4.433186	0.300124	-0.155981

$(HIO_3)_1(HIO_2)_1$

O	0.355973	-0.521508	-1.050174
I	1.868956	0.026732	-0.110950
O	1.189526	1.504723	0.662619
H	-0.413068	1.753072	0.113139
O	2.008772	-1.181880	1.179836

O	-1.302730	1.582360	-0.302646
I	-1.621763	-0.293799	-0.085600
O	-3.416405	0.104147	0.611644
H	-3.369237	0.498745	1.493812

$(HIO_3)_1(HIO_2)_2$

O	-0.346201	1.348132	1.575313
I	-0.417545	1.934145	-0.234402
O	1.185678	3.013860	-0.103219
H	1.935498	2.392342	-0.215038
O	2.629689	0.657079	-0.454154
I	2.245532	-1.006678	0.087918
O	1.090883	-0.760724	1.461980
H	0.268242	0.534164	1.593695
O	-2.326980	0.645053	-0.295770
I	-2.174331	-1.147232	0.194443
O	-1.334504	-1.920458	-1.321257
H	-0.308488	-1.804545	-1.237766
O	1.159059	-1.667247	-1.198232

$(HIO_3)_1(HIO_2)_3$

O	0.406134	0.424079	2.141354
I	1.197665	1.709480	0.966779

O	2.906104	1.609904	1.899357
H	3.336722	0.788186	1.592471
O	3.202162	-0.903575	0.590726
I	2.078283	-1.817919	-0.457239
O	0.511355	-1.649234	0.483486
H	0.483069	-0.443055	1.660106
O	-0.841527	1.754829	0.030003
I	-1.107930	0.923446	-1.645499
O	0.535273	1.622885	-2.431328
H	1.202574	0.910936	-2.326420
O	1.803516	-0.779396	-1.890815
O	-3.033912	0.098332	-0.615941
I	-2.778050	-0.762701	1.008206
O	-1.954971	-2.403262	0.472052
H	-0.963745	-2.224805	0.413593

$(HIO_3)_1(HIO_2)_4$

O	0.607551	-0.780146	-1.905855
I	0.873662	-2.067670	-0.533558
O	2.249091	-3.025188	-1.486403
H	3.058151	-2.475879	-1.399102
O	3.866649	-0.857187	-0.909994

I	3.547244	0.706789	-0.092361
O	1.949892	1.201579	-0.835579
H	1.143564	0.023974	-1.619875
O	-0.828067	-0.830370	0.550450
I	-0.262783	0.423007	1.896669
O	0.860814	-0.977698	2.698249
H	1.775266	-0.775894	2.405912
O	3.090181	0.263662	1.584992
O	-1.636527	1.738707	0.820328
I	-1.340521	2.002966	-1.029347
O	0.073613	3.322968	-0.760794
H	0.906554	2.810732	-0.737576
O	-2.912874	0.363417	-1.252741
I	-3.178662	-0.938641	0.056132
O	-5.091186	-1.156117	-0.308926
H	-5.580524	-0.393844	0.031525

$(HIO_3)_2(HIO_2)_1$

O	-2.567960	-0.900584	-0.538825
I	-1.085310	-1.499390	0.250948
O	-1.192805	-3.258522	-0.565415
H	-1.551925	-3.201131	-1.464397

O	0.216163	-0.971809	-0.954166
O	-0.860546	0.417787	1.119033
I	-0.980004	1.868870	-0.082398
O	-2.912221	1.998331	0.105381
H	-3.268415	1.155048	-0.232058
O	1.326593	1.522430	-0.158445
I	2.134321	-0.113577	-0.248253
O	3.852772	0.591944	0.288724
H	3.843496	0.831523	1.229079
O	1.802939	-0.943109	1.290168

$(HIO_3)_2(HIO_2)_2$

O	1.478938	3.019805	0.851720
I	-0.234593	2.244777	0.445624
O	0.144185	1.928078	-1.392498
H	0.686070	1.087371	-1.417246
O	-2.413822	1.530572	-0.143729
I	-3.286908	-0.049674	-0.398622
O	-2.130550	-1.047314	-1.365864
H	2.149354	2.298359	0.967669
O	1.158875	-2.025817	1.531517
I	-0.141793	-1.771642	0.136707

O	-1.416208	-2.925841	0.980379
H	-2.126733	-2.339433	1.325996
O	-3.213896	-0.823594	1.218265
I	3.325928	-0.176688	-0.482983
O	3.513953	-1.828975	0.184254
H	2.039910	-2.230256	1.134765
O	3.187402	0.927964	0.924307
O	1.582594	-0.241748	-1.057062

$(HIO_3)_2(HIO_2)_3$

O	2.991132	-0.938023	-1.272053
I	3.682212	0.417006	-0.291876
O	2.662872	1.828374	-0.728122
H	1.539081	2.891783	0.137239
O	3.119642	-0.036404	1.374225
O	0.778058	3.147088	0.707919
I	-0.068403	1.503929	1.220067
O	1.053845	1.150806	2.737947
H	1.872895	0.725096	2.390489
O	-1.780301	1.652778	-0.681528
I	-2.022751	0.430664	-1.984914
O	-0.996726	1.397524	-3.317809

H	-0.070598	1.507112	-3.046627
O	-0.762858	-0.818502	-1.737409
O	-0.493856	-2.880448	0.684750
I	0.961507	-2.039834	-0.236265
O	2.294523	-2.723686	0.973507
H	2.652925	-1.942149	1.442297
O	-3.319727	-0.649687	-0.596854
I	-3.265850	-0.407942	1.275329
O	-1.456807	-0.620379	1.703532
H	-0.908579	-2.160012	1.227707

$(HIO_3)_2(HIO_2)_4$

O	2.580753	-1.609739	1.417354
I	3.755729	-0.806980	0.307378
O	3.277545	-1.413850	-1.318233
H	2.652397	-0.211662	-2.522086
O	3.150656	0.901835	0.303254
O	1.913585	0.156530	-3.053118
I	0.393583	2.733297	-0.249830
O	2.163071	3.265712	-0.825307
H	2.755807	2.508293	-0.644571
O	-1.938132	-1.048557	-1.312310

I	-2.424877	-2.160465	0.021768
O	-1.552059	-3.661748	-0.814415
H	-0.797421	-3.349220	-1.359323
O	-1.234046	-1.969015	1.349384
O	-0.936072	0.656204	2.949078
I	0.495720	-0.375595	2.213314
O	1.821987	0.913803	2.785853
H	2.295337	1.197310	1.975601
O	-3.502177	-0.385930	0.768687
I	-3.333763	1.338013	0.028123
O	-1.719367	1.952940	0.755703
H	-1.316628	1.211065	2.220716
O	-0.166434	1.904944	-1.828451
I	0.301028	-0.314225	-2.115527
O	0.638124	-2.271633	-2.266633
H	1.497811	-2.432194	-1.834133

$(HIO_3)_3(HIO_2)_1$

O	-0.528685	0.014573	-1.692716
I	-2.234241	-0.613797	-1.490200
O	-2.025823	-2.203807	-0.703731
H	-1.356847	-2.909651	1.035325

O	-2.787187	0.488170	-0.167962
O	-0.924626	-2.726326	1.893007
I	-0.380494	-0.880768	1.816431
O	-2.057961	-0.195243	2.428112
H	-2.555896	0.071920	1.624609
O	1.976652	-1.760619	0.839274
I	2.726622	-1.072239	-0.629191
O	1.472664	-1.442921	-1.962492
H	0.602871	-0.858471	-1.857865
O	2.583747	0.697653	-0.463763
I	0.365914	2.407031	-0.008934
O	-1.459662	2.934223	-0.189106
H	-2.045138	2.140851	-0.146669
O	0.216799	1.483222	1.537281
O	1.038026	3.963989	0.466471

$(HIO_3)_3(HIO_2)_2$

O	3.093671	0.646523	-1.495733
I	3.888507	-0.476085	-0.312145
O	3.166362	0.103047	1.251008
H	2.094848	-0.834570	2.409899
O	3.107428	-2.062810	-0.581270

O	1.280545	-1.308920	2.687102
I	0.215713	-1.493883	1.100434
O	0.903376	-3.201086	0.580997
H	1.719292	-3.013603	0.063108
O	-0.537186	2.837569	-0.425957
I	1.002551	1.871082	-0.969922
O	2.072890	2.483376	0.492336
H	2.372761	1.685475	0.993234
O	-2.478211	0.998373	-0.777373
O	-0.757145	0.923253	1.526884
I	-2.038506	1.913211	0.755194
O	-3.507789	1.157531	1.744898
H	-3.574405	0.190660	1.583488
O	-1.073645	-1.483903	-1.110321
I	-2.851769	-1.212946	-1.099226
O	-3.363628	-3.015987	-1.562516
H	-3.097622	-3.645227	-0.872845
O	-3.280315	-1.358942	0.630371

$(HIO_3)_3(HIO_2)_3$

O	-2.152489	-1.447144	-1.731613
I	-2.415057	-2.455561	-0.263488

O	-0.779081	-3.094951	0.142590
H	0.201651	-3.035896	1.746394
O	-2.682315	-1.141553	0.970485
O	0.656046	-2.549717	2.466121
I	0.564520	-0.701401	1.970256
O	-0.964453	-0.350046	3.086716
H	-1.760603	-0.591821	2.568905
O	2.403925	-0.296718	-1.982428
I	0.542057	-0.635001	-1.962663
O	0.740917	-2.536988	-2.256337
H	0.602280	-2.970709	-1.393395
O	2.711511	1.252273	0.311715
O	2.456716	-1.519332	0.488322
I	3.547077	-0.256772	-0.207392
O	4.760477	-0.220960	1.322189
H	4.321033	0.066511	2.136541
O	0.297272	1.752618	-1.543312
I	0.595631	2.785234	-0.083436
O	-0.786304	3.929350	-0.242238
H	-2.247996	3.485776	-0.601569
O	-0.011402	1.755397	1.296296

O	-3.168849	3.053876	-0.659424
I	-2.941147	1.256287	-0.090514
O	-2.697763	1.620649	1.762271
H	-1.723297	1.774396	1.865907

$(HIO_3)_3(HIO_2)_4$

O	1.348018	1.509035	-1.787722
I	1.319645	3.011464	-0.774825
O	-0.449620	3.290338	-0.544147
H	-1.553419	3.385875	1.046878
O	1.867288	2.457030	0.852270
O	-1.922645	2.946309	1.840701
I	-1.300269	1.135959	1.743656
O	0.237687	1.498740	2.854028
H	0.889644	1.960922	2.277072
O	-2.923278	-0.599468	-1.899915
I	-1.180373	0.125170	-1.968474
O	-1.732566	1.849260	-2.648403
H	-1.645449	2.490453	-1.915574
O	-2.807123	-1.704829	0.679676
O	-3.338794	1.007548	0.293275
I	-4.024670	-0.622425	-0.084971

O	-5.202637	-0.671882	1.467624
H	-4.717915	-0.618372	2.305055
O	-0.473139	-2.072246	-1.179667
I	-0.311670	-2.613793	0.545359
O	1.392686	-3.236821	0.476247
H	2.662173	-2.492164	1.333273
O	-0.025322	-1.107557	1.505209
O	3.469405	-2.009589	1.655983
I	3.134591	-0.135863	1.545798
O	2.588559	0.004239	3.413135
H	1.615910	0.023758	3.447300
O	3.826575	-0.284622	-0.640257
I	2.553763	-0.938764	-1.836416
O	2.925062	-2.818695	-1.773403
H	2.383915	-3.177396	-1.027848

$(HIO_3)_4(HIO_2)_1$

O	0.229154	2.955018	-0.403424
I	-1.531656	2.664015	-0.702345
O	-1.641352	1.298534	-1.862055
H	-2.996934	0.004688	-2.040445
O	-2.096057	1.971656	0.878210

O	-3.535371	-0.738058	-1.688612
I	-2.780625	-1.106384	0.034058
O	-4.032458	-0.052046	1.045961
H	-3.594197	0.816378	1.167060
O	3.273496	-0.688580	-0.862867
I	3.465242	0.950337	-0.177776
O	2.273752	1.930041	-1.208948
H	1.328415	2.254893	-0.796621
O	2.746317	0.874943	1.458883
I	0.425043	-0.493952	1.903525
O	-0.105082	1.211261	2.519660
H	-0.859936	1.563837	1.971375
O	0.327134	-0.018162	0.114880
O	-1.128707	-1.346337	2.151047
I	0.800985	-1.892622	-1.041131
O	1.512209	-3.562423	-1.678883
H	1.394196	-4.255093	-1.008880
O	1.189210	-2.341290	0.688915
O	-0.931991	-2.346876	-1.172520

$(HIO_3)_4(HIO_2)_2$

O	-0.831708	-3.060643	0.146015

I	-2.093205	-2.478352	1.315537
O	-1.164918	-1.520223	2.518309
H	0.720713	0.076447	-3.079948
O	-3.010440	-1.252868	0.372721
O	0.916495	-0.876410	-3.187401
I	0.117532	-1.798754	-1.680280
O	-1.637948	-1.860745	-2.441764
H	-2.149888	-1.084146	-2.113416
O	1.129535	2.228966	1.327239
I	1.393827	0.689953	2.398118
O	-0.262487	0.819626	3.356299
H	-0.827097	0.082782	3.010287
O	1.835478	1.164382	-1.079545
O	-0.735599	0.762756	0.126847
I	0.314867	2.066922	-0.612451
O	-0.260129	1.633280	-2.429972
H	-1.229676	1.478988	-2.472194
O	3.527252	0.505046	1.161331
I	3.608704	-0.111211	-0.531735
O	5.259236	-1.074929	-0.285912
H	5.152542	-1.790255	0.361664

O	2.557992	-1.554315	-0.521185
I	-2.940328	1.375147	-0.212260
O	-4.715888	2.067001	-0.483161
H	-4.819840	2.389817	-1.392909
O	-2.072656	2.940296	-0.512251
O	-2.799320	0.632528	-1.841419

$(HIO_3)_4(HIO_2)_3$

O	-1.993241	-1.449460	-0.635692
I	-1.909914	-2.038555	1.117940
O	-0.302663	-2.840415	1.077085
H	1.146460	-2.700508	2.281079
O	-1.572841	-0.502264	1.998041
O	1.849952	-2.201161	2.745030
I	1.827782	-0.445064	1.973761
O	0.791277	0.304831	3.410732
H	-0.153120	0.179811	3.189523
O	2.385637	-0.865776	-2.343555
I	0.602583	-0.902099	-1.721107
O	0.501446	-2.841579	-1.747264
H	0.598039	-3.141440	-0.825067
O	3.580563	0.882910	-0.541160

O	3.065745	-1.763647	0.175867
I	4.028717	-0.796767	-1.008479
O	5.655369	-0.764869	0.070560
H	5.535353	-0.278312	0.900018
O	0.744278	1.474146	-1.691246
I	1.588795	2.656364	-0.598577
O	0.366679	3.970319	-0.555672
H	-1.268140	3.872538	-0.285214
O	1.353411	1.935556	1.059695
O	-2.167608	3.654945	0.098553
I	-1.989887	1.920390	0.854280
O	-0.987285	2.432725	2.403228
H	-0.037584	2.410554	2.135967
O	-4.383853	-1.035135	0.496462
I	-4.080446	-0.608357	-1.223458
O	-5.893339	-0.110430	-1.626249
H	-6.270102	0.446909	-0.925826
O	-3.385438	1.038937	-1.190855

$(HIO_3)_4(HIO_2)_4$

O	0.730609	1.604869	-1.332670
I	0.937128	2.605432	0.174103

O	-0.747510	3.257511	0.298920
H	-2.162306	3.170245	1.534273
O	0.947624	1.411025	1.532368
O	-2.813760	2.702447	2.102143
I	-2.367782	0.851572	1.926768
O	-1.078535	0.887832	3.346443
H	-0.214461	1.120975	2.942552
O	-3.477127	0.094723	-2.245502
I	-1.750754	0.783256	-1.906279
O	-2.156944	2.649004	-2.157608
H	-1.978517	3.084885	-1.299227
O	-3.464368	-1.619631	-0.039589
O	-4.207793	1.057337	0.220353
I	-4.719307	-0.463134	-0.611739
O	-6.009325	-0.975019	0.760035
H	-5.619838	-1.029250	1.645512
O	-0.726673	-1.579149	-1.359204
I	-0.936151	-2.613766	0.123943
O	0.748316	-3.265024	0.243925
H	2.169533	-3.203410	1.480773
O	-0.954879	-1.450241	1.508589

O	2.819641	-2.742062	2.055071
I	2.364151	-0.890634	1.911454
O	1.071769	-0.958095	3.326826
H	0.209339	-1.186556	2.916121
O	3.479335	-0.049850	-2.245420
I	1.753466	-0.746356	-1.918885
O	2.162906	-2.607197	-2.200228
H	1.987771	-3.057265	-1.348690
O	3.462720	1.627907	-0.013050
I	4.719090	0.481189	-0.601748
O	6.005916	0.969664	0.781441
H	5.615739	1.004070	1.667590
O	4.208221	-1.054088	0.203078

REFERENCES

(1) McGrath, M. J.; Olenius, T.; Ortega, I. K.; Loukonen, V.; Paasonen, P.; Kurtén, T.; Kulmala, M.; Vehkamäki, H., Atmospheric Cluster Dynamics Code: a flexible method for solution of the birth-death equations. *Atmos. Chem. Phys.* **2012**, *12*, (5), 2345-2355.

(2) Georgievskii, Y.; Klippenstein, S. J., Long-range transition state theory. *J. Chem. Phys.* **2005**, *122*, (19), 194103.

(3) Kürten, A.; Jokinen, T.; Simon, M.; Sipilä, M.; Sarnela, N.; Junninen, H.; Adamov, A.; Almeida, J.; Amorim, A.; Bianchi, F.; Breitenlechner, M.; Dommen, J.; Donahue, N. M.; Duplissy, J.; Ehrhart, S.; Flagan, R. C.; Franchin, A.; Hakala, J.; Hansel, A.; Heinritzi, M.; Hutterli, M.; Kangasluoma, J.; Kirkby, J.; Laaksonen, A.; Lehtipalo, K.; Leiminger, M.; Makhmutov, V.; Mathot, S.; Onnela, A.; Petäjä, T.; Praplan, A. P.; Riccobono, F.; Rissanen, M. P.; Rondo, L.; Schobesberger, S.; Seinfeld, J. H.; Steiner, G.; Tomé, A.; Tröstl, J.; Winkler, P. M.; Williamson, C.; Wimmer, D.; Ye, P.; Baltensperger, U.; Carslaw, K. S.; Kulmala, M.; Worsnop, D. R.; Curtius, J., Neutral molecular cluster formation of sulfuric acid-dimethylamine observed in real time under atmospheric conditions. *Proc. Natl. Acad. Sci. U.S.A.* **2014**, *111*, (42), 15019-15024.

(4) Halonen, R.; Zapadinsky, E.; Kurtén, T.; Vehkamäki, H.; Reischl, B., Rate enhancement in collisions of sulfuric acid molecules due to long-range intermolecular forces. *Atmos. Chem. Phys.* **2019**, *19*, (21), 13355-13366.

(5) He, X.-C.; Tham, Y. J.; Dada, L.; Wang, M.; Finkenzeller, H.; Stolzenburg, D.; Iyer, S.; Simon, M.; Kürten, A.; Shen, J.; Rörup, B.; Rissanen, M.; Schobesberger, S.;

Baalbaki, R.; Wang, D. S.; Koenig, T. K.; Jokinen, T.; Sarnela, N.; Beck, L. J.; Almeida, J.; Amanatidis, S.; Amorim, A.; Ataei, F.; Baccarini, A.; Bertozzi, B.; Bianchi, F.; Brilke, S.; Caudillo, L.; Chen, D.; Chiu, R.; Chu, B.; Dias, A.; Ding, A.; Dommen, J.; Duplissy, J.; Haddad, I. E.; Carracedo, L. G.; Granzin, M.; Hansel, A.; Heinritzi, M.; Hofbauer, V.; Junninen, H.; Kangasluoma, J.; Kemppainen, D.; Kim, C.; Kong, W.; Krechmer, J. E.; Kvashin, A.; Laitinen, T.; Lamkaddam, H.; Lee, C. P.; Lehtipalo, K.; Leiminger, M.; Li, Z.; Makhmutov, V.; Manninen, H. E.; Marie, G.; Marten, R.; Mathot, S.; Mauldin, R. L.; Mentler, B.; Möhler, O.; Müller, T.; Nie, W.; Onnela, A.; Petäjä, T.; Pfeifer, J.; Philippov, M.; Ranjithkumar, A.; Saiz-Lopez, A.; Salma, I.; Scholz, W.; Schuchmann, S.; Schulze, B.; Steiner, G.; Stozhkov, Y.; Tauber, C.; Tomé, A.; Thakur, R. C.; Väisänen, O.; Vazquez-Pufleau, M.; Wagner, A. C.; Wang, Y.; Weber, S. K.; Winkler, P. M.; Wu, Y.; Xiao, M.; Yan, C.; Ye, Q.; Ylisirniö, A.; Zauner-Wieczorek, M.; Zha, Q.; Zhou, P.; Flagan, R. C.; Curtius, J.; Baltensperger, U.; Kulmala, M.; Kerminen, V.-M.; Kurtén, T.; Donahue, N. M.; Volkamer, R.; Kirkby, J.; Worsnop, D. R.; Sipilä, M., Role of iodine oxoacids in atmospheric aerosol nucleation. *Science* **2021**, *371*, (6529), 589-595.

(6) He, X.-C.; Iyer, S.; Sipilä, M.; Ylisirniö, A.; Peltola, M.; Kontkanen, J.; Baalbaki, R.; Simon, M.; Kürten, A.; Tham, Y. J.; Pesonen, J.; Ahonen, L. R.; Amanatidis, S.; Amorim, A.; Baccarini, A.; Beck, L.; Bianchi, F.; Brilke, S.; Chen, D.; Chiu, R.; Curtius, J.; Dada, L.; Dias, A.; Dommen, J.; Donahue, N. M.; Duplissy, J.; El Haddad, I.; Finkenzeller, H.; Fischer, L.; Heinritzi, M.; Hofbauer, V.; Kangasluoma, J.; Kim, C.; Koenig, T. K.; Kubečka, J.; Kvashnin, A.; Lamkaddam, H.; Lee, C. P.; Leiminger, M.;

Li, Z.; Makhmutov, V.; Xiao, M.; Marten, R.; Nie, W.; Onnela, A.; Partoll, E.; Petäjä, T.; Salo, V.-T.; Schuchmann, S.; Steiner, G.; Stolzenburg, D.; Stozhkov, Y.; Tauber, C.; Tomé, A.; Väisänen, O.; Vazquez-Pufleau, M.; Volkamer, R.; Wagner, A. C.; Wang, M.; Wang, Y.; Wimmer, D.; Winkler, P. M.; Worsnop, D. R.; Wu, Y.; Yan, C.; Ye, Q.; Lehtinen, K.; Nieminen, T.; Manninen, H. E.; Rissanen, M.; Schobesberger, S.; Lehtipalo, K.; Baltensperger, U.; Hansel, A.; Kerminen, V.-M.; Flagan, R. C.; Kirkby, J.; Kurtén, T.; Kulmala, M., Determination of the collision rate coefficient between charged iodic acid clusters and iodic acid using the appearance time method. *Aerosol. Sci. Tech.* **2021**, *55*, (2), 231-242.

(7) Khanniche, S.; Louis, F.; Cantrel, L.; Černušák, I., Computational study of the $I_2O_5 + H_2O = 2HOIO_2$ gas-phase reaction. *Chem. Phys. Lett.* **2016**, *662*, 114-119.

(8) Rong, H.; Liu, J.; Zhang, Y.; Du, L.; Zhang, X.; Li, Z., Nucleation mechanisms of iodic acid in clean and polluted coastal regions. *Chemosphere* **2020**, *253*, No.126743.

(9) Kumar, M.; Saiz-Lopez, A.; Francisco, J. S., Single-Molecule Catalysis Revealed: Elucidating the Mechanistic Framework for the Formation and Growth of Atmospheric Iodine Oxide Aerosols in Gas-Phase and Aqueous Surface Environments. *J. Am. Chem. Soc.* **2018**, *140*, (44), 14704-14716.

(10) Zhang, S.; Li, S.; Ning, A.; Liu, L.; Zhang, X., Iodous acid – a more efficient nucleation precursor than iodic acid. *Phys. Chem. Chem. Phys.* **2022**, *24*, (22), 13651-13660.

(11) Gómez Martín, J. C.; Lewis, T. R.; Blitz, M. A.; Plane, J. M. C.; Kumar, M.; Francisco, J. S.; Saiz-Lopez, A., A gas-to-particle conversion mechanism helps to explain atmospheric particle formation through clustering of iodine oxides. *Nat. Commun.* **2020**, *11*, (1), 4521.

(12) Dunne, E. M.; Gordon, H.; Kürten, A.; Almeida, J.; Duplissy, J.; Williamson, C.; Ortega, I. K.; Pringle, K. J.; Adamov, A.; Baltensperger, U.; Barmet, P.; Benduhn, F.; Bianchi, F.; Breitenlechner, M.; Clarke, A.; Curtius, J.; Dommen, J.; Donahue, N. M.; Ehrhart, S.; Flagan, R. C.; Franchin, A.; Guida, R.; Hakala, J.; Hansel, A.; Heinritzi, M.; Jokinen, T.; Kangasluoma, J.; Kirkby, J.; Kulmala, M.; Kupc, A.; Lawler, M. J.; Lehtipalo, K.; Makhmutov, V.; Mann, G.; Mathot, S.; Merikanto, J.; Miettinen, P.; Nenes, A.; Onnela, A.; Rap, A.; Reddington, C. L. S.; Riccobono, F.; Richards, N. A. D.; Rissanen, M. P.; Rondo, L.; Sarnela, N.; Schobesberger, S.; Sengupta, K.; Simon, M.; Sipilä, M.; Smith, J. N.; Stozkhov, Y.; Tomé, A.; Tröstl, J.; Wagner, P. E.; Wimmer, D.; Winkler, P. M.; Worsnop, D. R.; Carslaw, K. S., Global atmospheric particle formation from CERN CLOUD measurements. *Science* **2016**, *354*, (6316), 1119-1124.

(13) Xie, H.-B.; Elm, J.; Halonen, R.; Myllys, N.; Kurten, T.; Kulmala, M.; Vehkamaki, H., Atmospheric Fate of Monoethanolamine: Enhancing New Particle Formation of Sulfuric Acid as an Important Removal Process. *Environ. Sci. Technol.* **2017**, *51*, (15), 8422-8431.
